# A Microbial Lipid‐ATP Synthase Axis Fuels NK Cell Antitumor Activity

**DOI:** 10.1002/advs.202520095

**Published:** 2026-04-13

**Authors:** Kaiyuan Yu, Xinyu Sun, Wanxia Ma, Jianming Yang, Xuan Sun, Lisong Zhang, Yumeng Liu, Tianshu Ren, Qi Wang, Jingyu Wang, Xiao Li, Xianping Peng, Liu Yang, Junqiang Lv, Zhi Yao, Zhi‐Song Zhang, Quan Wang

**Affiliations:** ^1^ Tianjin Institute of Immunology, Key Laboratory of Immune Microenvironment and Disease (Ministry of Education), School of Basic Medical Sciences, Tianjin Medical University, State Key Laboratory of Experimental Hematology Chinese Academy of Medical Sciences and Peking Union Medical College Tianjin China; ^2^ State Key Laboratory of Medicinal Chemical Biology and College of Pharmacy, Tianjin Key Laboratory of Molecular Drug Research Nankai University Tianjin China; ^3^ Institute of Medicinal Biotechnology, Chinese Academy of Medical Sciences and Peking Union Medical College State Key Laboratory of Experimental Hematology Beijing China

**Keywords:** ATP5F1A, antitumor immunity, NK cells, outer membrane vesicles, sphingosine

## Abstract

The gut microbiota influences systemic immunity and cancer through inter‐organ communication, but OMV‐mediated mechanisms remain unclear. Here, we uncover a previously unrecognized role of *Bacteroides intestinalis* in restraining extra‐intestinal tumor growth via OMVs enriched in sphingosine (SP), a bioactive lipid that directly binds to ATP5F1A—a subunit of the mitochondrial ATP synthase—to enhance NK cell function. This microbial lipid‐ATP synthase interaction augments mitochondrial efficiency, reduces reactive oxygen species (ROS) production, and potently upregulates IFN‐γsecretion in NK cells, driving increased cytotoxicity and tumor infiltration. Remarkably, OMVs from *B. intestinalis* or SP administration greatly inhibit murine tumor growth, while their combination with anti‐PD‐1 therapy enhances systemic antitumor immunity. This study establishes the specific immune activation ability for gut microbial OMVs and highlights microbiota‐derived lipid‐based immunotherapies.

## Graphical abstract

This study focuses on the mechanism by which gut microbiota‐derived outer membrane vesicles (OMVs) regulate NK cell antitumor activity. *B. intestinalis* is identified to decrease extra‐intestinal tumor growth via its OMVs enriched in sphingosine (SP). SP directly binds to ATP5F1A, enhances mitochondrial function, and boosts NK cell cytotoxicity to strengthen systemic antitumor immunity.

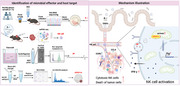

## 1 Introduction

The close relationship between gut microbiota and tumor progression has been widely recognized [1, 2, 3]. This microbial community not only regulates the progression of intestinal malignancies but also exerts significant influence on extra‐intestinal tumor development and therapeutic outcomes [4, 5, 6]. Among the various intercellular communication mechanisms, outer membrane vesicles (OMVs)—biologically active nanovesicles secreted by many Gram‐negative gut bacteria—have emerged as critical mediators of microbial‐host interactions [7, 8, 9]. Emerging evidence suggests that specific bacterial OMVs exhibit antitumor properties and show promise as next‐generation cancer immunotherapeutics [10, 11, 12]. Despite these insights, the role of gut microbiota‐derived OMVs in extra‐intestinal tumor progression remains to be characterized.

Natural killer (NK) cells serve as critical mediators of innate antitumor immunity through their rapid, antigen‐independent recognition and cytotoxic elimination of malignant cells [13, 14]. Despite extensive evidence of the gut microbiota systemic effects on tumors, the mechanisms underlying its regulation of NK cell‐mediated immunity remain poorly understood, and the interactions between gut microbiota‐derived OMVs and NK cells in cancer immunosurveillance have not been characterized.

Chronic or severe inflammation promotes tumorigenesis via multiple mechanisms, whereas several studies indicate that gut inflammation and microbiota alteration induced by radiotherapy, chemotherapy, or cell dysfunction can enhance the antitumor immune response in extra‐intestinal tumors, implying that transient mild inflammation may exert antitumor effects through gut microbiota and immune system remodeling [15, 16, 17]. Here, mouse and human gut microbiota influenced by mild colitis were demonstrated to suppress extra‐intestinal tumor growth. We further identified sphingosine (SP) from gut bacterial OMVs as a critical mediator that enhances NK cell mitochondrial function through direct interaction with ATP synthase F1 subunit alpha (ATP5F1A), ultimately augmenting antitumor immunity against extra‐intestinal malignancies. Our findings reveal a previously uncharacterized mechanism of direct communication between gut microbiota‐derived OMVs and NK cells in tumor modulation, and propose a novel strategy for microbiota‐based cancer immunotherapy.

## 2 Results

### 2.1 Bacteroides Intestinalis Suppresses Extra‐Intestinal Tumor Progression

We administered mice with 1% dextran sulfate sodium (DSS) to induce mild colitis and evaluated its effects on subcutaneous LLC1 lung cancer and B16 melanoma models (Figure S1A). DSS treatment resulted in significant suppression of subcutaneous tumor growth compared with water‐treated controls (Figure S1B–E). Compared with severe colitis, the degree of intestinal inflammation induced by 1% DSS‐induced colitis is significantly milder (Figure S1F–J). To investigate the role of gut microbiota in this effect, we depleted the intestinal microbiota using a broad‐spectrum antibiotic cocktail (Abx) for 7 days prior to DSS administration (Figure S1K,L), and observed that the tumor‐inhibitory effect of DSS was completely abrogated (Figure S1M,N).

**FIGURE 1:**
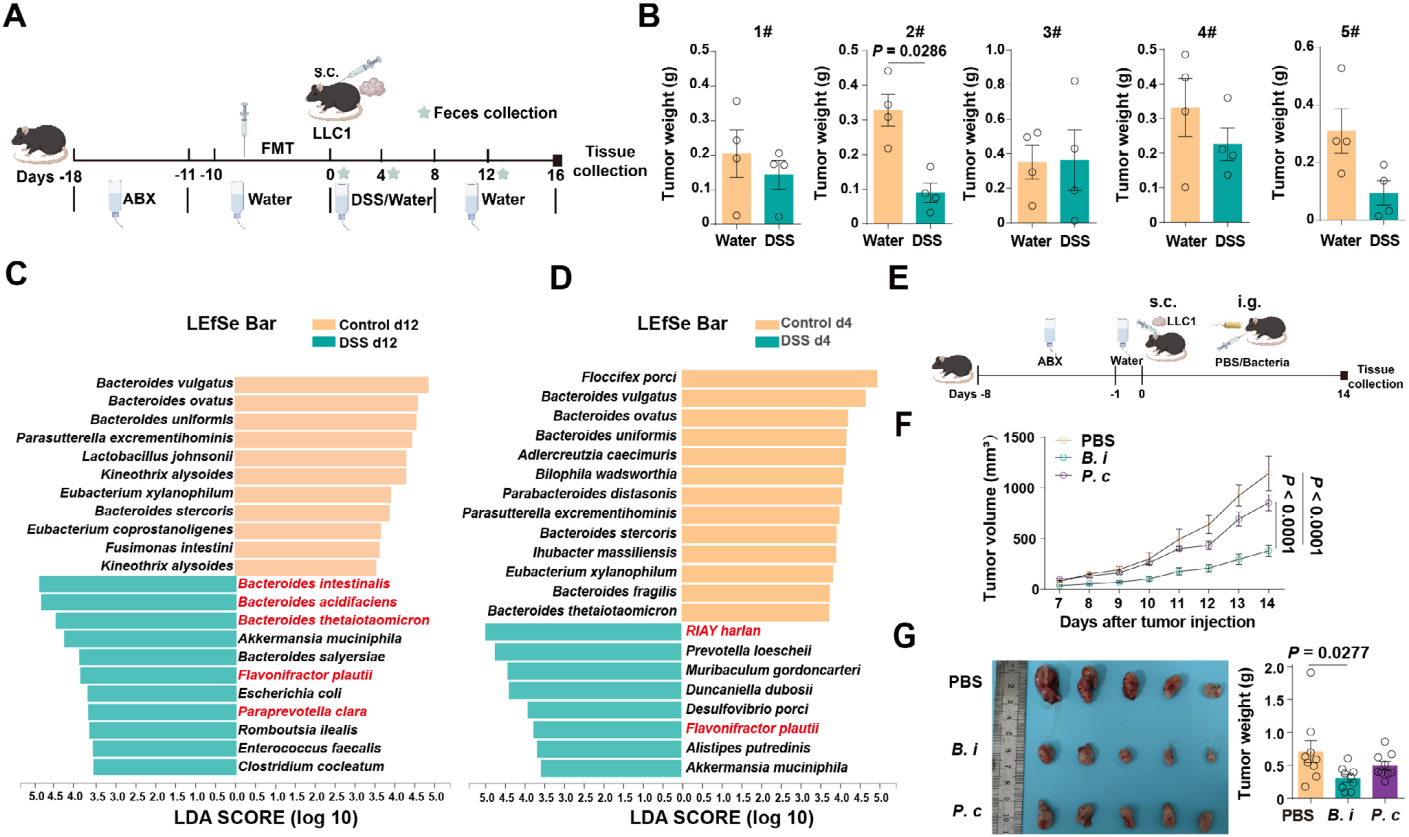
***B. intestinalis* inhibits extra‐intestinal tumor growth. (A**) Schematic of FMT strategy followed by subcutaneous LLC1 implantation and tumor progression analysis. Mice received 7 days of ampicillin/vancomycin/neomycin/metronidazole (ABX) and 1 day of water treatment, then were gavaged daily with stool from healthy donors for 10 days. Subsequent 8 days of 1% DSS and LLC1 inoculation on day 0 were applied. Asterisks mark fecal collection time points. (**B**) Tumor volumes in DSS‐ and water‐treated groups after FMT from five healthy donors (*n*  =  4 mice/group). (**C, D**) Bar charts of LDA scores for gut microbiota in two mouse groups on day 4 (**C**) or day 12 (**D**) post‐FMT with feces from healthy donor 2#. (**E**) Schematic of single‐strain treatment (5×10^8^ CFU *B. intestinalis* (*B. i*), *P. clara* (*P. c*), or PBS) following 7 days of ABX and subcutaneous LLC1 inoculation on day 0. (**F, G**) Tumor growth curves (**F**) and final tumor weights (**G**) of LLC1 tumors in C57BL/6J mice treated as indicated (*n* = 9 mice/group). All data are presented as mean ± s.e.m. Statistical analysis: two‐tailed Mann‐Whitney test (**B**), two‐way ANOVA followed by Sidak's comparison test (**F**), or one‐way ANOVA followed by Dunnett's comparison test (**G**).

To determine if human intestinal microbiota exhibits similar antitumor properties, we performed fecal microbiota transplantation (FMT) from healthy donors into mice followed by DSS treatment. Transplanted DSS‐treated microbiota demonstrated extra‐intestinal tumor suppression comparable to mouse models (Figure 1A,B). Focusing on the most effective donor (Donor #2), we used 16S rRNA gene sequencing to identify enriched bacterial strains after DSS treatment (Figure 1C,D). Subsequent single‐strain colonization experiments with six significantly enriched and isolated strains revealed that only *Bacteroides intestinalis* administration resulted in significant tumor growth inhibition (Figure 1E–G and Figure S1O–R), identifying *B. intestinalis* as the specific tumor‐suppressive member of the mild colitis‐modulated microbiota.

### 2.2 B. Intestinalis Inhibits Tumors Growth Through Enhanced Cytotoxic NK Cell Infiltration

**FIGURE 2:**
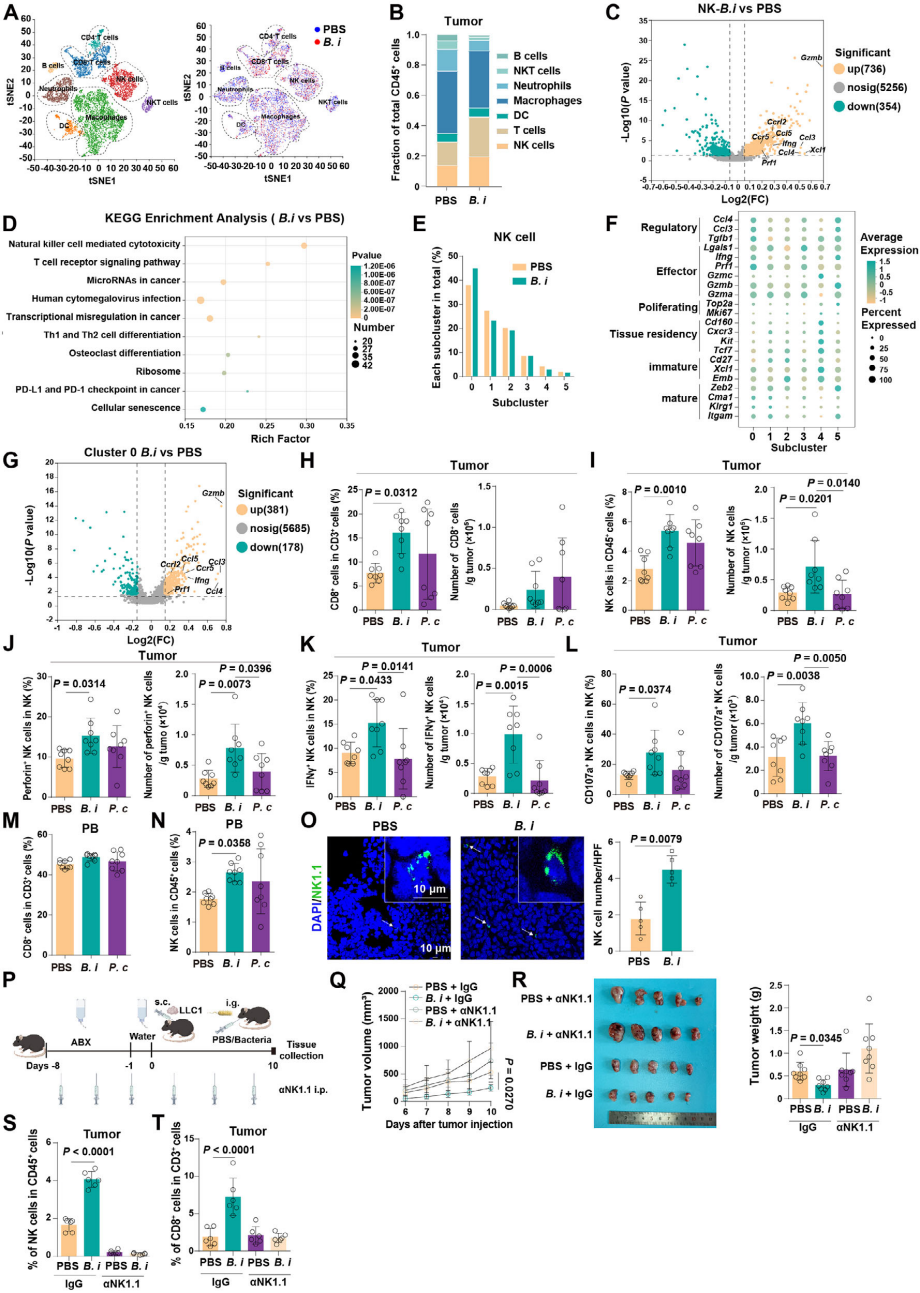
***B. intestinalis* inhibits tumor progression by promoting NK cell function. (A**) tSNE plot of scRNA‐seq clusters of tumor‐infiltrating immune cells sorted from the model in Figure 1E. (**B**) Proportional distribution of CD45^+^ cell clusters. (**C**) Volcano plots of differentially expressed genes in NK cells between the two groups. (**D**) KEGG pathway enrichment analysis of upregulated genes in the *B. intestinalis* group NK cell subcluster. (**E**) Proportions of NK cell subclusters in the two groups. (**F**) Dot plots of distinct NK cell signature gene expression across subclusters. (**G**) Volcano plots of differentially expressed genes in NK cluster 0 between the two groups. **H,L**, Proportions and counts of CD8^+^ T, total NK, and cytotoxic NK cells in LLC1 tumors from the model in Figure 1E (*n* = 8 mice/group). (**M, N**) Proportions of CD8^+^ T and total NK cells in peripheral blood (PB) from the model in Figure 1E (*n* = 8 mice/group). (**O**) Immunofluorescence analysis of NK cell infiltration in tumors from the model in Figure 1E. Scale bar: 10 µm (*n* = 5 mice/group). (**P**) Schematic of tumor‐suppressive model with single‐strain bacteria after NK depletion. Mice were injected intraperitoneally with anti‐NK1.1 or IgG, then gavaged daily with 5×10^8^ CFU *B. intestinalis* or PBS after 7 days of ABX and 1 day of water. LLC1 cells were subcutaneously inoculated on day 0. (**Q**, **R**) Tumor growth curves (**Q**) and tumor weights (**R**) of LLC1 tumors in C57BL/6J mice treated as indicated (*n *= 8 mice/group). **S,T**, Proportions of NK and CD8^+^ T cells in LLC1 tumors from C57BL/6J mice (*n* = 6 mice/group). All data are presented as mean ± s.e.m. Statistical analysis: one‐way ANOVA followed by Tukey's multiple comparisons test (**H**–**N, R–T**), two‐tailed Mann‐Whitney test (**O**), or two‐way ANOVA followed by Sidak's comparison test (**Q**).

Considering the critical role of the tumor immune microenvironment (TIME) in cancer progression, we performed single‐cell RNA sequencing (scRNA‐seq) of CD45^+^ immune cells infiltrated in LLC1 tumors following *B. intestinalis* colonization. Eight distinct leukocyte populations were identified (Figure 2A), and notably, *B. intestinalis* colonization significantly increased tumor infiltration of NK and CD8^+^ T cells compared to the control (Figure 2B).

Focusing on NK cell, we observed enhanced chemotactic migration and cytotoxic activity following *B. intestinalis* treatment (Figure 2C,D). scRNA‐seq revealed six NK cell subclusters, with Cluster 0 being the largest and showing marked expansion in *B. intestinalis*‐colonized mice (Figure 2E,F and Figure S2A,B), which exhibited elevated expression of cytotoxic mediators, chemokine, and chemokine receptors (Figure 2G). Analysis of CD8^+^ T cells revealed enhanced cytotoxic potential, chemotactic activity, and antigen processing capacity after *B. intestinalis* treatment (Figure S2C,D). Clustering based on gene expression patterns identified four CD8^+^ T cell subclusters, with Cluster 0 (effector phenotype) showing increased abundance in *B. intestinalis* recipients (Figure S2E–I).

To validate these findings, we characterized the tumor microenvironment of mice treated with *B. intestinalis*, *Paraprevotella clara* (another DSS‐enriched species), or PBS using flow cytometry (Figure 1E). *B. intestinalis* uniquely enhanced NK and CD8^+^ T cell infiltration (Figure 2H,I and Figure S1S) and improved NK cell effector functions (Figure 2J–L). In contrast, no significant changes in CD4^+^ T cell infiltration were observed (Figure S2J). Peripheral blood analysis showed increased NK cell frequencies but no significant changes in CD4^+^ or CD8^+^ T cells (Figure 2M,N and Figure S2K). Importantly, *B. intestinalis* colonization did not alter immune cell proportions in colon tissues (Figures S1T and S2L–N). Immunofluorescence staining confirmed increased NK cells and Gzmb^+^ cytotoxic NK cells in *B. intestinalis*‐treated tumors (Figure 2O and Figure S2O–Q).

To establish causality, we depleted NK cells with NK‐neutralizing antibody before *B. intestinalis* treatment (Figure 2P and Figure S2R). This abrogated the antitumor effects of *B. intestinalis* (Figure 2Q,R), demonstrating NK cell dependency. Significantly, NK cell depletion also eliminated the *B. intestinalis*‐induced enhancement of CD8+ T cell (Figure 2S,T), suggesting potential crosstalk between NK and CD8+ T cell responses.

### 2.3 *B. intestinalis*‐Derived OMVs Enhance NK Cell Cytotoxic Immunity

Given the potential for gut bacteria to translocate to tumor sites via the bloodstream and modulate tumor progression [18, 19, 20], we investigated whether *B. intestinalis* could colonize extra‐intestinal tumors. Oral gavage of *B. intestinalis* induced colonization in the colon without altering its length, tissue architecture, or mucosal barrier integrity (Figure S3A–D). Crucially, no bacterial colonization was detected in subcutaneous tumor tissues (Figure S3E,F), suggesting that the antitumor effect of *B. intestinalis* is indirect. We further assayed heat‐killed (HK) *B. intestinalis* and its culture supernatant, which exhibited no effect on tumor growth (Figure S3G–L), which indicates that the observed effect depends on viable bacteria cells.

**FIGURE 3:**
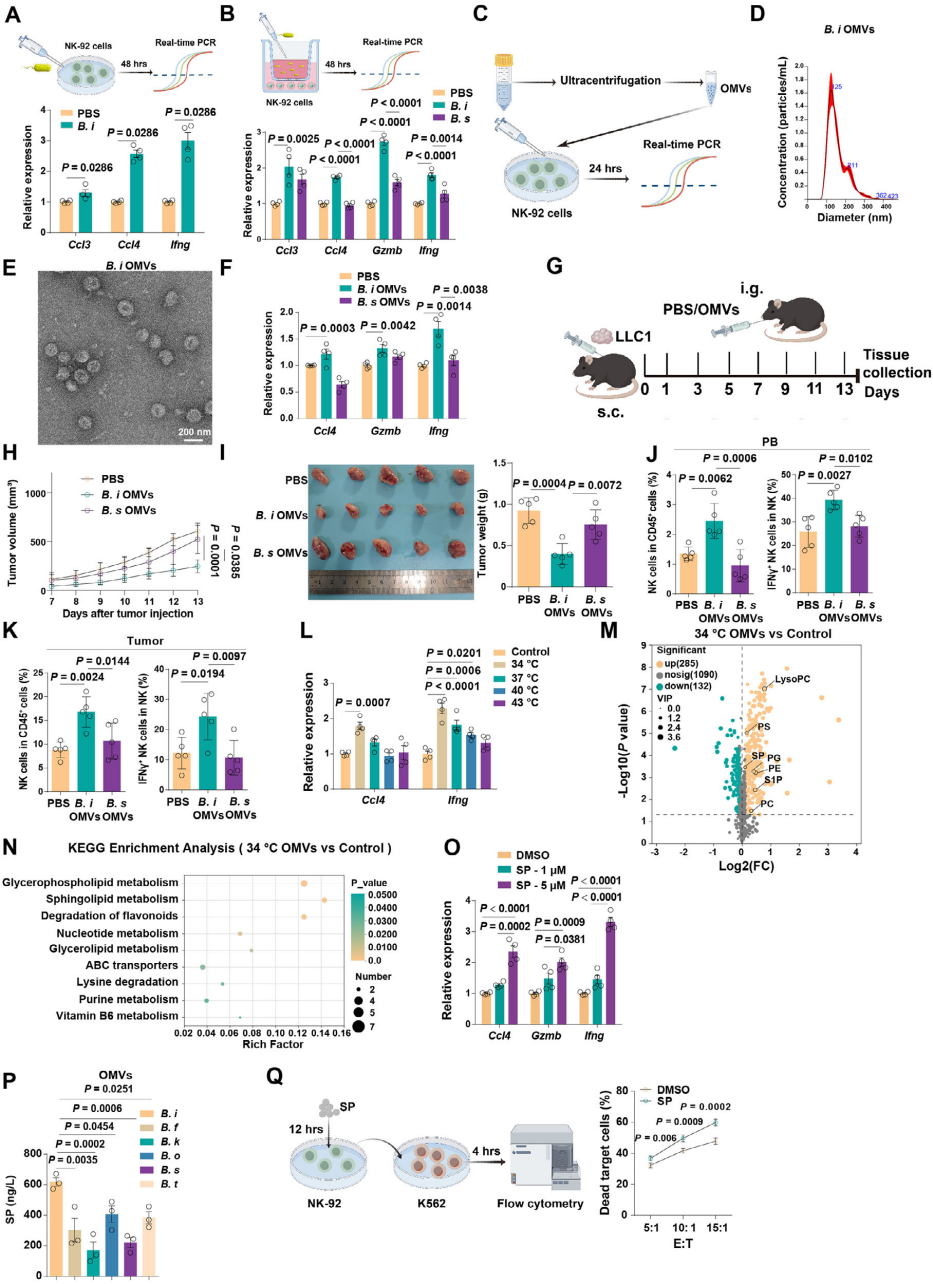
***B. intestinalis*‐derived OMVs promote the antitumor function of NK cells. (A, B**) NK‐92 cells were stimulated with *B. intestinalis* (MOI = 200, 48 h) (**A**) or cultured in agar‐coated transwell inserts with *B. intestinalis* in the upper chamber and NK‐92 in the lower chamber (**B**). mRNA levels of *Ccl3, Ccl4*, and *Ifng* were analyzed by qRT‐PCR (*n* = 4). (**C‐F)**, Schematic of OMV effects on NK‐92 functional genes (**C**). Nanoparticle tracking analysis (NTA) (**D**) and representative TEM images (**E**) of *B. intestinalis* OMVs (Scale bar: 200 nm). Functional analysis of NK‐92 stimulated with *B. intestinalis* OMVs (50 µg/mL, 24 h) (**F**) for *Ccl3*, *Ccl4*, and *Ifng* mRNA levels (*n* = 4). (**G**) Schematic of tumor‐suppressive model with *B. intestinalis* OMVs. Mice received OMVs (40 µg) or PBS every other day after LLC1 implantation. (**H**, **I)** Tumor growth curves (**H**) and tumor weights (**I**) of LLC1 tumors in mice treated as indicated (*n* = 5 mice/group). (**J, K**) Proportions of NK and IFN‐γ^+^ NK cells in peripheral blood (**J**) and tumors (**K**) from C57BL/6J mice treated with OMVs or PBS (*n* = 5 mice/group). (**L**) NK‐92 cells were stimulated with *B. intestinalis* OMVs cultured at different temperatures, followed by qRT‐PCR analysis of *Ccl4* and *Ifng* mRNA levels (*n* = 4). (**M, N**) Metabolite analysis of OMVs from *B. intestinalis* cultured at 34°C versus GAM broth (*n* = 3). Volcano plots (**M**), KEGG pathway enrichment of upregulated metabolites (**N**). (**O**) NK‐92 cells were stimulated with Sphingosine (SP) (12 h) for qRT‐PCR analysis of *Ccl4, Gzmb*, and *Ifng* mRNA levels(*n* = 4). (**P**) SP levels in OMVs from different Bacteroides species. (**Q**) K562 cell killing by NK‐92 cells cultured with SP (5 µm, 12 h) or DMSO. Proportion of PI‐positive K562 cells by flow cytometry (*n* = 4). All data are presented as mean ± s.e.m. Statistical analysis: two‐tailed Mann–Whitney U test (**A**), one‐way ANOVA followed by Tukey's multiple comparisons test or Sidak's multiple comparisons test (**B**, **F**, **I**–**L**, **O**–**P**), two‐way ANOVA followed by Sidak's comparison test (**H**), or two‐tailed unpaired Student's t test (**Q**).

To understand the mechanisms underlying the notable NK cell changes, we further analyzed the interplay between *B. intestinalis* and NK cells. Co‐culture experiments revealed that *B. intestinalis‐*exposed NK‐92 cells (a widely used human natural killer cell line) exhibited increased expression of genes associated with cytotoxicity (*Ifng*) and chemokine production (*Ccl3*, *Ccl4*) (Figure 3A). In contrast, HK *B. intestinalis* and bacterial supernatant failed to induce similar effects (Figure S4A,B), supporting the hypothesis that active bacterial products but not metabolites or secreted proteins are critical. A transwell co‐culture system was employed to examine the effects of active factors from *B. intestinalis* on NK cells. This system demonstrated that bacterial‐derived products significantly enhanced *Gzmb*, *Ifng*, *Ccl3*, and *Ccl4* expression (Figure 3B), consistent with the hypothesis that extracellular signaling, rather than direct contact, drives NK cell activation.

To identify the key immunomodulatory component, we extracted OMVs from *B. intestinalis* and *B. stercorirosoris* and tested their effects on NK cells in vitro (Figure 3C). No significant differences in OMV size distribution were observed between the two species (Figure 3D,E and Figure S4C,D). However, OMVs from *B. intestinalis* selectively stimulated *Ifng* expression in NK cells after uptake (Figure S4E and Figure 3F). Oral gavage of *B. intestinalis*‐derived OMVs (*B. i* OMVs) confirmed their ability to cross the intestinal barrier, accumulate in blood NK cells, and reach subcutaneous tumors (Figure S4F–I).

Functionally, *B. i* OMVs exhibited antitumor activity in vivo (Figure 3G–I). Flow cytometric analysis revealed increased proportions of NK cells and cytotoxic NK cells in both peripheral blood and tumor tissues following OMV administration (Figure 3J, K and Figure S4J–M). Notably, these effects were absent in mice treated with OMVs from *B. stercorirosoris*, further validating the specificity of *B. i* OMVs.

Together, these findings suggest that *B. i* OMVs serve as functional mediators of antitumor immunity by enhancing NK cell infiltration and cytotoxic activity.

### 2.4 Sphingosine in OMVs From *B. intestinalis* Mediates NK Cell Cytotoxic Activity

Environmental factors influence bacterial adaptation by modulating the composition of OMVs [21, 22]. To identify functional components of *B. i* OMVs, we isolated OMVs under different cultural conditions and tested their effects on NK cell activation. Temperature significantly modulated OMV functionality, with OMVs from *B. intestinalis* cultured at 34°C showing enhanced induction of *Ifng* expression in NK cells compared to those cultured at other temperatures (Figure 3L and Figure S5A, B).

To determine the molecular basis of this effect, we further examined lysed *B. i* OMVs and treated them with protease K and DNase. Neither treatment diminished OMV‐induced *Ifng* upregulation in NK cells (Figure S5C), suggesting that protein/DNA components are not essential for this activity and perhaps a key role for OMV‐associated metabolites mediates these effects. LC‐MS/MS analysis of OMVs revealed distinct metabolic profiles between 34°C‐ and 43°C‐cultured *B. i* OMVs and the control (Figure 3M and Figure S5D, E). KEGG pathway enrichment analysis highlighted significant upregulation of glycerophospholipid and sphingolipid metabolism pathways in 34°C‐derived OMVs (Figure 3N and Figure S5F, G). Among the seven upregulated metabolites in these pathways, sphingosine (SP) exhibited the most pronounced effect on *Ifng* expression in NK‐92 cells (Figure 3O and Figure S5H–M). Significantly elevated SP levels were also observed in *B. i* OMVs versus other *Bacteroides* species (Figure 3P), and higher SP content was confirmed in *B. i* OMVs cultured at 34°C compared to 43°C (Figure S5N). Functional validation showed that SP accumulated in the cytoplasm of NK cells within a short period of time and significantly enhanced NK cell cytotoxicity against tumor cells, independent of NK cell proliferation (Figure 3Q and Figure S5O–Q). Notably, SP did not alter the expression of cytotoxic or chemokine‐related genes in CD8^+^ T cells, highlighting its specificity for NK cell activation (Figure S5R). SP also significantly enhances the regulatory effect of NK cells on the cytotoxic effector function of CD8+ T cells (Figure S5S).

### 2.5 Sphingosine Promotes NK Cell‐Mediated Antitumor Activity

**FIGURE 4:**
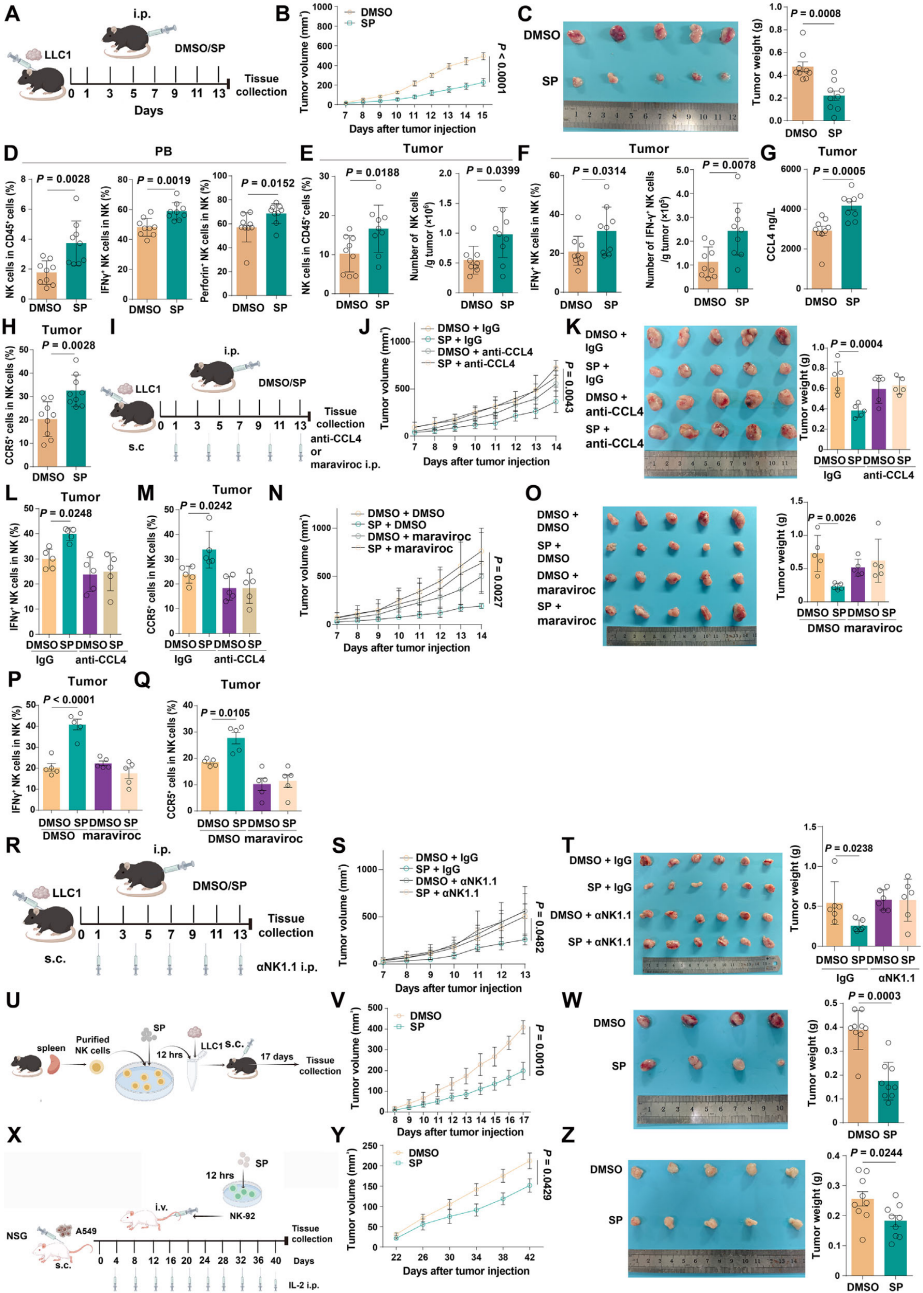
**The critical role of Sphingosine in NK cell‐mediated inhibition of tumor growth. (A–H**) Schematic of LLC1 tumor model with SP treatment. Mice were intraperitoneally injected with SP (10 mg/kg) or DMSO every other day after tumor implantation (**A**). Tumor growth curves (**B**) and tumor weights (**C**) in DMSO‐ or SP‐treated groups (*n* = 9 mice/group). Proportions and counts of total NK and IFN‐γ^+^ NK cells in peripheral blood (**D**) and tumors (**E**, **F**), CCR5^+^ NK cells in tumors (**H**) or CCL4 levels in the tumors (**G**) from DMSO‐ or SP‐treated mice (*n* = 9 mice/group). (**I–Q**) Schematic of LLC1 tumor model using maraviroc (a CCR5 antagonist) or CCL4 neutralizing antibody. After tumor inoculation, mice were intraperitoneally injected with SP (10 mg/kg) or DMSO every two days, and with isotype control, maraviroc (10 mg/kg) or CCL4 neutralizing antibody (1 mg/kg) every three days (**I**). Tumor growth curves (**J**) and tumor weights (**K**) in DMSO‐ or SP‐treated mice with CCL4 depletion (*n* = 5 mice/group). Proportions of IFN‐γ^+^ and CCR5^+^ NK cells in tumors (**L, M**). Tumor growth curves (**N**) and tumor weights (**O**) in DMSO‐ or SP‐treated mice with CCR5 depletion (*n* = 5 mice/group). Proportions of CCR5^+^ and IFN‐γ^+^ NK cells in tumors (**P**, **Q**). (**R–T**) Schematic of LLC1 tumor model with NK cell depletion. Mice received SP (10 mg/kg) or DMSO every other day and NK neutralizing antibody (200 µg) every three days after tumor implantation (**R**). Tumor growth curves (**S**) and tumor weights (**T**) in DMSO‐ or SP‐treated mice with NK neutralization (*n* = 6 mice/group). (**U–W**) Schematic of LLC1 tumor model with pre‐treated NK cells. Mice were subcutaneously co‐inoculated with LLC1 cells and splenic NK cells pre‐treated with DMSO or SP (5 µm) for 12 h (**U**). Tumor growth curves (**V**) and tumor weights (**W**) in DMSO‐ or SP‐treated NK cell groups (*n* = 9 mice/group). (**X–Z**) Schematic of human A549 tumor model. A549 cells were subcutaneously injected into NSG mice, and DMSO‐ or SP‐treated NK92 cells (5 µm, 12 h) were transferred via tail vein (**X**). Tumor growth curves (**Y**) and tumor weights (**Z**) in DMSO‐ or SP‐treated NK92 cell groups (*n* = 9 mice/group). All data are presented as mean ± s.e.m. Statistical analysis: two‐way ANOVA followed by Sidak's comparison test (**B**, **J**, **N**, **S**, **V, Y**), two‐tailed Mann‐Whitney test (**C**–**H**, **W**, **Z**), or one‐way ANOVA followed by Sidak's multiple comparisons test (**K‐Q, T**).

To evaluate the in vivo antitumor effects of SP, we administered it intraperitoneally to mice and observed significant inhibition of subcutaneous tumor growth (Figure 4A–C). After intraperitoneal injection, a significant increase in SP levels was observed in the plasma of the mice (Figure S6A). Concurrently, SP treatment increased both the proportion and absolute number of NK cells and cytotoxic NK cells in peripheral blood and tumor tissues (Figure 4D–F and Figure S6B–E). Single‐cell RNA sequencing (scRNA‐seq) revealed that elevated expression of *Ccl4* and *Ccr5* in NK cells in the *B. intestinalis*‐treated group (Figure S6F), aligning with SP directly upregulated *Ccl4* expression in NK cells (Figure 3O). These findings led to the hypothesis that SP‐induced CCL4 drives CCR5^+^ NK cell infiltration into tumors. Experimental validation confirmed that tumor tissues from SP‐treated mice exhibited significantly elevated intratumoral CCL4 levels and increased frequencies of CCR5^+^ NK cells (Figure 4G,H), while SP had no effect on *Ccr5* expression in NK cells (Figure S6G). Inhibition of the CCR5 receptor or CCL4 not only abrogated the tumor growth‐inhibitory activity of SP but also eliminated its effect on CCR5+ and IFN‐γ+ NK cells (Figure 4I–Q and Figure S6H). To directly assess NK cell dependence, we performed neutralization experiments and observed complete abrogation of SP's antitumor effects (Figure 4R–T and Figure S6I). Functional studies demonstrated that SP‐treated mouse splenic NK cells exhibited enhanced tumor‐suppressive activity in mice (Figure 4U–W), and this effect was recapitulated in the model where SP‐treated NK‐92 cells showed improved inhibition of human lung cancer cells in NSG mice (Figure 4X–Z). Serum SP levels were significantly higher in both mild and severe colitis mouse groups than in the control group, yet tumor growth was markedly slowed only in the mild colitis group, which may be attributed to the severe inflammatory response counteracting the anti‐tumor effect of SP (Figure S6J–N).

Next, we evaluated the therapeutic synergy between SP and checkpoint blockade therapy in a murine model of subcutaneous lung cancer (Figure S7A). While monotherapy with SP or anti‐PD‐1 treatment reduced LLC1 tumor growth, the combination synergistically suppressed tumor burden (Figure S7B,C). Furthermore, SP enhanced anti‐PD‐1‐mediated CD8^+^ T cell antitumor activity, potentially through crosstalk between activated NK cells and CD8^+^ T cells (Figures S7D and S7E). It has been reported that *B. intestinalis* is enriched in faecal samples of responders compared to non‐responders among NSCLC patients receiving anti‐PD‐1 treatment [23]. Analyses of several public datasets demonstrate that fecal *B. intestinalis* levels are probably associated with immunotherapy response in cancer patients (NCBI: PRJNA1023797, European Nucleotide Archive: ERP134027) [24, 25], and serum SP concentrations are significantly higher in healthy individuals than in colorectal cancer patients (Figure S7F, G) [26].

To confirm SP's role as the critical mediator in *B. i* OMVs, we engineered an SP‐deficient *B. intestinalis* mutant by CRISPR‐mediated deletion of serine palmitoyltransferase (SPT), the rate‐limiting enzyme in SP biosynthesis [27, 28] (Figure S6O, P). Quantification revealed a significant reduction in SP content within OMVs of the *B. intestinalis ^ΔSPT^* mutant, though SP was not completely eliminated, possibly because SPT is the major, but not exclusive, biosynthetic pathway for SP in *B. intestinalis*. (Figure S7H). Functionally, these OMVs showed impaired capacity to upregulate *Ifng* expression in NK cells (Figure S7I). Consistent with in vitro results, the tumor‐suppressive activity of *B. intestinalis^ΔSPT^* OMVs was markedly attenuated compared to wild‐type OMVs (Figure 3G and Figure S7J, K). Analysis of immune cell populations confirmed the functional dependency on SP: the *B. intestinalis^ΔSPT^* group exhibited significantly reduced frequencies of NK cells and IFN‐γ^+^ NK cells in both peripheral blood and tumor tissues compared to wild‐type controls (Figure S7L,M), and no significant changes were observed in other immune cell types (Figure S6Q–T).

These findings demonstrate that the tumor‐suppressive activity of *B. intestinalis* OMVs is largely dependent on SP, which enhances NK cell cytotoxicity and promotes tumor infiltration through CCL4‐CCR5 axis.

### 2.6 Sphingosine Interacts With ATP5F1A to Enhance NK Cell Function

**FIGURE 5:**
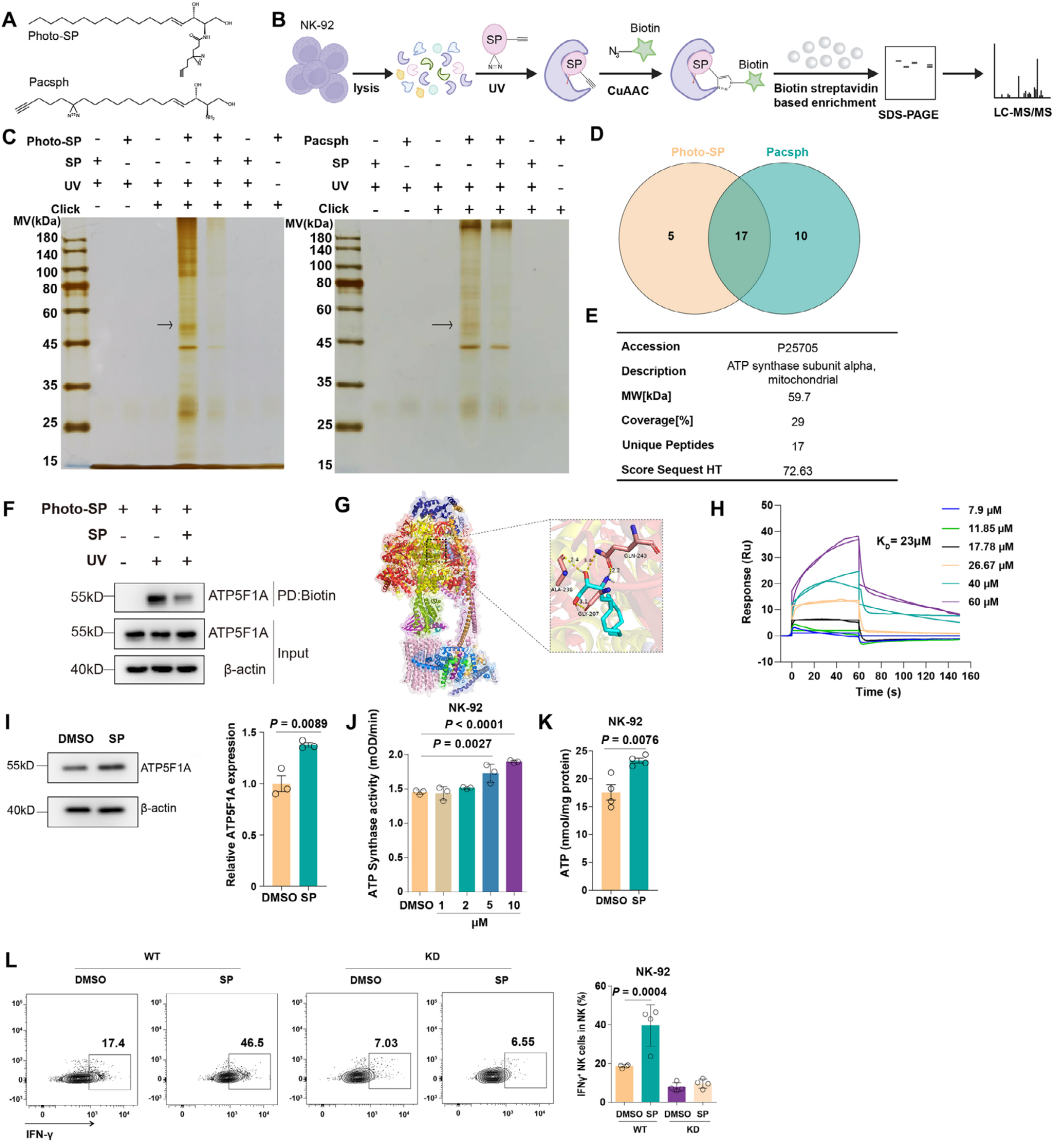
**Sphingosine targets ATP5F1A to enhance NK cell function. (A**) Chemical structures of clickable photoaffinity probes: Photo‐SP and Pacsph. (**B**) Schematic workflow for target protein capture and identification in NK‐92 cells using Photo‐SP and Pacsph. (**C**) Silver staining of proteins interacting with 200 µm Photo‐SP or 50 µm Pacsph in NK‐92 cells under the indicated conditions. Arrows indicate bands enriched by UV irradiation and click reaction. (**D**) Venn diagram of target proteins significantly enriched by Photo‐SP and Pacsph. (**E**) ATP synthase subunit alpha (ATP5F1A) was identified by mass spectrometry from enriched protein bands captured by both Photo‐SP and Pacsph. (**F**) Pull‐down analysis of ATP5F1A using Photo‐SP in NK‐92 cells. (**G**) Molecular docking model of SP binding to ATP synthase. Right panel: enlarged view of interactions within the α‐subunit. (**H**) SPR analysis of ATP5F1A–SP interaction. SP was passed at serial dilutions (60–7.9 µm) to immobilized ATP5F1A. RU, response units. (**I–K**) Functional assays of ATP5F1A in NK‐92 cells ATP5F1A expression (*n* = 3) (**I**), F1F0‐ATPase activity (*n* = 3) (**J**) and ATP analysis (*n* = 4) (**K**) after treatment with DMSO or SP for 9 h. (**L**) Wildtype (WT) and ATP5F1A‐koockdown (KD) NK‐92 cells were stimulated with DMSO or SP (6 µm) for 9 h. Intracellular IFN‐γ levels were analyzed by flow cytometry (*n* = 4). All data are presented as mean ± s.e.m. Statistical analysis: two‐tailed Mann‐Whitney test (**I**, **K**) or one‐way ANOVA followed by Sidak's multiple comparisons test (**J**, **L**).

To identify the SP‐targeted protein in NK cells mediating cytotoxicity, we synthesized two chemical reporter molecules: a photoaffinity‐labeled SP derivative with diazirine and alkyne tags at the head group (Photo‐SP) and ordered a tail‐modified SP analog (Pacsph) (Figure 5A). After incubating these probes with NK‐92 cell lysates, a click reaction was performed using azide‐modified Biotin probes, and the products were enriched with Streptavidin‐agarose. Silver staining and mass spectrometry analysis revealed specific enrichment of a 50 kDa protein band in both groups compared to controls (Figure 5B,C). The overlapping subset identified included ATP5F1A, a structural subunit of ATP synthase (Figure 5D,E).

To confirm SP‐ATP5F1A interaction, we conducted pull‐down assays using cell lysates, demonstrating the binding between SP and ATP5F1A (Figure 5F). Subsequent molecular docking studies using the CB‐Dock2 platform revealed that SP shows high binding affinity for the ATP5F1A subunit of human ATP synthase, forming hydrogen bonds with GLY207, ALA236, GLN243 (predicted with PyMol software) (Figure 5G). Surface plasmon resonance (SPR) experiments further validated stable direct binding of SP to ATP5F1A (Figure 5H). To validate the critical role of these predicted binding sites, we generated a recombinant ATP5F1A mutant protein containing the G207A, A236V, and Q243A substitutions and assessed its binding to SP using SPR under identical assay conditions. Notably, the binding affinity of SP for the mutant protein was significantly reduced (Figure S7N), supporting that these residues contribute to the SP–ATP5F1A interaction interface. To elucidate the mechanism of SP‐induced ATP5F1A upregulation, we first examined *Atp5f1a* mRNA levels, which remained unchanged (Figure S7O). Cycloheximide (CHX) treatment revealed that SP sustained ATP5F1A protein stability, ruling out enhanced de novo synthesis (Figure S7P). Furthermore, MG132 treatment confirmed proteasomal involvement (Figure S7Q). Thus, SP stabilizes ATP5F1A by suppressing proteasome‐dependent degradation.

Functionally, the protein content of ATP5F1A is higher in SP‐treated NK‐92 cells (Figure 5I). We also measured ATP synthase enzyme activity and ATP production, finding significant increases in both parameters following SP treatment (Figure 5J,K). The ability of SP to stimulate IFN‐γ production was abrogated by ATP5F1A knockdown, indicating that ATP5F1A plays a key role in SP‐mediated enhancement of NK cell cytotoxicity (Figure 5L and Figure S7R).

### 2.7 SP‐ATP5F1A Interaction Regulates Mitochondrial Function to Augment NK Cells Cytotoxicity

**FIGURE 6:**
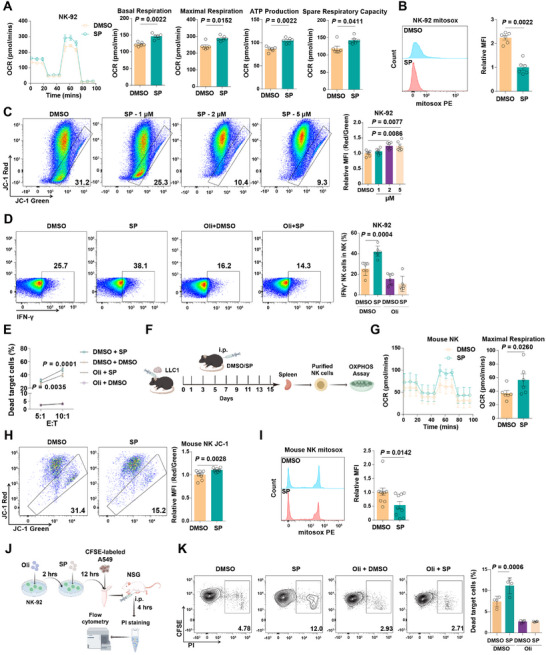
**Sphingosine regulates mitochondrial function to promote NK cell cytotoxicity. (A–C)** Functional analysis of mitochondrial activity. Cellular oxygen consumption rate (OCR) levels (**A**), intracellular reactive oxygen species (ROS) levels (**B**), and mitochondrial membrane potential (**C**) were measured in NK‐92 cells treated with SP or DMSO (*n* = 6). (**D**, **E**) NK‐92 cells were pretreated with oligomycin (2 µm, 2 h), then stimulated with DMSO or SP (5 µm) for 12 h. Intracellular IFN‐γ levels (**D**) and K562 cell killing (**E**) were analyzed by flow cytometry (*n* = 6). (**F–I**) Schematic illustration of the effects of SP on mitochondrial function in NK cells in vivo. Mice received intraperitoneal injections of SP (10 mg/kg) or DMSO every other day after tumor implantation. Splenic NK cells were isolated for analysis (**F**). OCR levels (**G**), ROS levels (**H**), and mitochondrial membrane potential (**I**) were measured (*n* = 6 mice/group). (**J, K)** Schematic illustration of in vivo cytotoxicity assay using CFSE‐labeled A549 cells and NK‐92 adoptive transfer in NSG mice. NK‐92 cells were pretreated with oligomycin (2 µm, 2 h), then stimulated with DMSO or SP (5 µm) for 12 h (**J**). Flow cytometry analysis of NK‐92‐mediated cytotoxicity in vivo using CFSE‐labeled A549 cells as targets and PI staining to detect dead cells (**K**) (*n* = 4). All data are presented as mean ± s.e.m. Statistical analysis: two‐tailed Mann‐Whitney test (**A**, **B**, **G**–**I**) or one‐way ANOVA followed by Sidak's multiple comparisons test (**C**–**E, K**).

Given the well‐established role of oxidative phosphorylation (OXPHOS) in enhancing NK cell migration and cytotoxicity [29, 30, 31], and the direct activation of ATP5F1A by SP (Figure 5), we evaluated mitochondrial function in SP‐treated NK cells. Oxygen consumption rate (OCR) assays revealed that SP significantly enhanced aerobic respiration (Figure 6A). Furthermore, SP reduced basal reactive oxygen species (ROS) levels and elevated mitochondrial membrane potential (Figure 6B,C). Functional validation demonstrated that SP‐induced IFN‐γ production in NK‐92 cells was abrogated by oligomycin, an ATP synthase inhibitor (Figure 6D). Notably, oligomycin also eliminated the tumor cell‐killing activity of SP‐treated NK cells (Figure 6E).

To confirm these effects in vivo, we isolated splenic NK cells from SP‐treated tumor‐bearing mice for mitochondrial function analysis (Figure 6F). The SP‐treated group exhibited increased maximal oxidative capacity, higher mitochondrial membrane potential, and reduced ROS levels compared to the control (Figure 6G–I). Moreover, we tested the cytotoxicity of NK‐92 cells treated with SP and ATP synthase inhibitor‐oligomycin against A549 tumor cells in NSG mice (Figure 6J), and results demonstrated that SP‐induced ATP synthase activation was essential for enhanced tumor cell killing (Figure 6K).

Collectively, these findings establish a direct functional link between SP‐ATP5F1A complex, mitochondrial function, and NK cell antitumor activity.

## 3 Discussion

Emerging evidence indicates that the intestinal microbiota modulates extra‐intestinal tumor development through three distinct mechanisms as follows: the gut microbiota regulates the migration of immune cells between the intestine and systemic sites, disrupting immune compartmentalization and shaping distant antitumor responses [32, 33, 34]; intestinal bacteria produce soluble metabolites and signaling molecules that systemically affect immune responses throughout the body [6, 35, 36]; certain gut bacteria can translocate to tumor microenvironments to directly influence tumor‐associated immune responses [37, 38]. Notably, the specific bioactive molecules within gut microbiota‐derived OMVs and their host targets remain undefined. Our findings identify a previously uncharacterized mechanism by which *B. intestinalis*‐derived OMVs modulate extra‐intestinal tumor progression through sphingosine‐mediated activation of NK cell mitochondrial function via interacting with ATP5F1A. This discovery expands the mechanistic understanding of gut microbiota in cancer immunotherapy and highlights OMVs as an important class of microbial effectors.

The interplay between intestinal inflammation and extra‐intestinal tumors is context‐dependent and complex. While clinical studies have shown that inflammatory bowel disease (IBD) is associated with increased risks of various extra‐intestinal tumors [39, 40, 41, 42], colitis paradoxically has also been reported to enhance antitumor immunity through immune activation [15, 43, 44, 45]. This apparent paradox may stem from the fact that the severity of inflammation and its effects on gut microbiota‐immune interactions are critical determinants. The elevated SP levels observed in patients with severe ulcerative colitis, combined with our finding of increased *B. intestinalis* abundance (an SP‐producing bacterium) in mild inflammation, suggest that inflammatory environments may promote the survival of SP‐producing bacteria. The reason may be as follows: (1) Mild DSS‐induced inflammation stimulates intestinal epithelial cells to secrete mucins [46]. *B. intestinalis* encodes glycoside hydrolases and polysaccharide lyases that efficiently degrade mucin‐derived polysaccharides into utilizable carbon and nitrogen sources, conferring a competitive nutritional advantage [47]. (2) Mild inflammation generates reactive oxygen species (ROS), which *B. intestinalis* can scavenge via endogenous peroxidases to tolerate oxidative stress. In contrast, most commensal bacteria are ROS‐sensitive with growth inhibition, further favoring *B. intestinalis* enrichment [48]. Notably, despite consistent SP elevation across colitis severities, tumor growth was inhibited in mild but promoted in severe models. Taken together, we propose that SP may function as an antitumor agent in low‐grade inflammation by modulating immune activity; however, this effect could be overwhelmed by the carcinogenic consequences of severe chronic inflammation. The role of SP in tumor suppression under different inflammatory contexts requires further in‐depth studies to determine its possible therapeutic relevance in diverse clinical scenarios.

Sphingolipid metabolism in eukaryotic cells plays a critical role in tumor progression and immune cell function [49, 50, 51]. Sphingolipids are also key constituents of bacterial OMVs, and *Bacteroides* strains are major members of the human gut commensal microbiota. Recent studies demonstrate that sphingolipid in *Bacteroides*‐derived OMVs modulate host anti‐inflammatory and immune tolerance pathways, underscoring their immunomodulatory potential [52]. In our study, the prioritization of ATP synthase ATP5F1A as the target of SP was based on proteomic enrichment patterns and cross‐probe reproducibility. Specifically, we focused on the ∼50 kDa region enriched by both Photo‐SP and Pacsph, selecting proteins that met the following criteria: identified by both probes (overlapping hits), indicating higher‐confidence enrichment; molecular weight matching ∼50 kDa and ranking high in the prioritization score; strong biological relevance to core cellular bioenergetics. ATP5F1A emerged as a high‐priority candidate in this overlap set. Importantly, ATP5F1A is directly linked to mitochondrial energy metabolism. Existing studies have demonstrated that enhanced OXPHOS function in NK cells increases ATP production to support IFN‐γ secretion [53, 54]. This study demonstrates that SP directly binds ATP5F1A, stabilizing its level by inhibiting proteasome‐mediated degradation. Consequently, ATP synthase activity, OXPHOS function, and NK cell cytotoxicity are enhanced. Further investigation is warranted to fully elucidate the underlying molecular mechanisms.

In the present study, we demonstrate that *B. intestinalis* promotes CD8^+^ T cell infiltration in an NK‐dependent manner, as NK depletion abolished this effect. Additionally, SP‐primed NK cells enhanced CD8^+^ T cell function. Thus, SP acts indirectly on CD8^+^ T cells but via NK cells, pending further validation of the underlying complex cellular interactions. Previous studies have shown that SP induces mitochondrial depolarization by binding to cardiolipin, thereby triggering apoptosis in pancreatic cancer cells [55]. This indicates that SP can promote tumor cell death by inhibiting mitochondrial function. Our findings differ from these reports, as SP exhibited distinct effects on mitochondrial function in different cell types. Such discrepancies may arise from context‐dependent roles of SP across cell populations. Future research should investigate its specific effects on mitochondrial function in immune and tumor cells.

In the harsh gastrointestinal environment, gastric acid and bile salts significantly reduce the viability of orally administered probiotics [56], while gastrointestinal peristalsis accelerates bacterial elimination [57]. Therefore, delivery barriers remain a key limitation for the clinical translation of probiotics. In the present study, evidence of *B. intestinalis* colonization was observed in murine intestines (Figure S3A); however, the stability of this colonization was not assessed. To advance the clinical application of *B. intestinalis*, optimized delivery strategies are needed to minimize bacterial loss in the gastrointestinal tract and improve intestinal colonization efficiency and functional stability.

Direct clinical validation remains a limitation. Although *B. intestinalis* and its metabolite SP were derived from human feces and demonstrated anti‐tumor efficacy preclinically, clinical evidence relies on public dataset reanalysis. These analyses associate fecal *B. intestinalis* with immunotherapy response and indicate reduced serum SP in colorectal cancer patients. However, quantification of fecal SP levels in patients with extraintestinal tumors (e.g., lung cancer or melanoma) and their associations with clinical outcomes, such as overall survival, progression‐free survival, immune therapy response rates, and intratumoral NK cell infiltration, remains to be determined. The definitive clinical significance and translational potential require further rigorous validation in large‐scale, multi‐center, and well‐characterized clinical cohort studies.

In summary, our findings demonstrate for the first time that SP enriched in *B. intestinalis* OMVs, directly enhances NK cell tumor‐killing activity through a specific interaction with ATP5F1A. This discovery establishes the first mechanistic link between bacterial sphingolipid signaling and host antitumor immunity, and expands the current understanding of gut bacterial OMVs as key regulators of tumor‐immune system interactions.

## 4 Methods

### 4.1 Mice

Wild‐type female C57BL/6J mice (6–8 weeks of age) were purchased from SPF Biotechnology Co., Ltd. NOD‐Cg‐*Prkdc^scid^Il2r^gem1cya^*/Cya (NSG) mice were obtained from Cyagen Biosciences. All mice were maintained under specific pathogen‐free conditions at the Department of Laboratory Animal Science and Technology, Tianjin Medical University. All experiments with mice were approved by the Animal Care and Use Committee, Tianjin Medical University (TMUaMEC 2024034).

### 4.2 Human Fecal Samples

Fresh fecal samples were collected from five healthy volunteers (age range: 20–26 years; 3 males, 2 females). The study was approved by the Ethics Committee of Tianjin Beichen Hospital, China (approval no. 2023032908), and informed consent was obtained from all participants.

### 4.3 Bacterial Strains and Growth Conditions

Seven strains, namely Bacteroides intestinalis, Bacteroides acidifaciens, Bacteroides thetaiotaomicron, Flavonifractor plautii, Paraprevotella clara, RIAY harlan, and Flavonifractor plautii were isolated from the feces of mice on day 4 and day 12 after fecal microbiota transplantation. Bacteroides strains were cultured anaerobically at 37°C in GAM broth (Hope Bio‐Technology, HB8518) supplemented with 1% Vitamin K1 (Hope Bio‐Technology, HB0310b)‐Hemin (Hope Bio‐Technology, HB0310a) Solution.

### 4.4 Cells

Human NK cell line NK‐92 cells were cultured in NK‐92 medium [α‐MEM medium (Thermo Fisher Scientific, C12571500BT) containing 12.5% fetal bovine serum (FBS, Gibco, A5256701), 12.5% horse serum (Pricella, 164215), 0.2 mm inositol (Sigma, I7508), 0.1 mm β‐mercaptoethanol (Thermo Fisher Scientific, 21985023), 0.02 mm folic acid (Sigma, I7508)], containing 100 U/mL recombinant human IL‐2 (Peprotech, 96‐200‐02‐50). Mouse lung cancer cell lines LLC1, human CML cells K562, and human lung cancer cell lines A549 were cultured in RPMI1640 medium (BasalMedia, L220KJ) plus 10% FBS. Mouse melanoma cells B16‐F10 were cultured in DMEM medium plus 10% FBS. PK136 cells were cultured in DMEM medium plus 10% FBS. Cell lines were purchased from ATCC. Spleen CD8^+^ cells of mice were sorted using a FACSAria Cell Sorter (BD Biosciences). CD8^+^ cells were stimulated with 100 U/mL recombinant mouse IL‐2 (Peprotech, 212‐12‐20), 1 µg/mL anti‐mouse CD3ε (Biolegend, 100340), and 5 µg/mL anti‐mouse CD28 (Biolegend, 122021) in RPMI1640 medium with 10% FBS, 10 mm HEPES (Sigma, 83264), 100 µm non‐essential amino acids (NEAA), 100 U/mL penicillin, 100 µg/mL streptomycin (Life‐iLab, AC03L332), and 50 µm β‐mercaptoethanol (Thermo Fisher Scientific, 21985023). Spleen CD8^+^ cells of mice were sorted using a FACSAria Cell Sorter (BD Biosciences). Spleen NK cells of mice were sorted using the NK cell isolation kit (Biolegend, 480049) in RPMI1640 medium containing 10% FBS, 1000 U/mL recombinant mouse IL‐2 (Peprotech 212‐12‐20), and 20 µg/mL recombinant mouse IL‐15 (Peprotech, 210‐15‐10). Mycoplasma contamination was routinely tested. All cells were maintained at 37°C, 21% O_2_, and 5% CO_2_.

### 4.5 Mouse Model of Depletion of Gut Microbiota

To deplete the gut microbiota, mice were given a cocktail of antibiotics [ABX, ampicillin (A610028‐0025, 1 g/L), neomycin (A610366‐0025, 1 g/L), metronidazole (A600633‐0025, 1 g/L), and vancomycin (A600983‐0001, 500 mg/L), SangonBiotech] added in water, followed by oral gavage daily for seven days. Water containing ABX was refreshed every 3 days.

### 4.6 Fecal Microbiota Transplantation (FMT)

Two grams of healthy people feces were homogenized in 5 mL of sterile Brain Heart Infusion (Hopebio, HB8297‐5) containing 30% glycerine (Solarbio, G8190) and 0.1% L‐cysteine (Macklin, L804954) with vortex mixing and then centrifuged at 800 g for 5 min. The supernatant was filtered through a 70 µm filter to remove large particles under anaerobic conditions. Before FMT, mice were given ABX added to water, followed by oral gavage daily for seven days. Mice were gavaged daily with 200 µl of fresh fecal solution for ten days after one day of water treatment.

### 4.7 Purification of NK Cell Neutralizing Antibodies

Each female BALB/c mouse was intraperitoneally injected with 500 µL pristane (Solarbio, T7330). 7 days after injection, 1.5 × 10^7^ PK136 cells were inoculated intraperitoneally. 10 days later, ascites fluid was collected and stored at −80°C. On the night before antibody purification, the ascites fluid was placed on ice and thawed overnight at 4°C. Subsequently, centrifugation was performed at 10 000 rpm and 4°C for 15 min. The supernatant was transferred to a new centrifuge tube on ice and filtered through a 0.22 µm filter into another new centrifuge tube. The filtrate was diluted with PBS or serum‐free medium and subjected to protein purification using a chromatographic column.

### 4.8 Animal Model

In the DSS‐inhibited extra‐intestinal tumor model, acute colitis was induced using 1% (mild) or 3% (severe) DSS (MP Biomedicals, 0216011090) for 8 days. After tumor inoculation in mice, 1% DSS was replaced with autoclaved water. To investigate the role of gut microbiota in DSS‐inhibited tumor growth, mice were treated with ABX for 7 days followed by combined ABX and DSS treatment. Mice were subjected to FMT after intestinal microbiota depletion with ABX. Following tumor implantation, the mice were treated with water or 1% DSS, with fecal samples collected on days 0, 4, and 12. For single‐strain tumor inhibition experiments, mice were treated with ABX, provided water for 1 day, then inoculated with tumors, followed by gavaged with 200 µL liquid bacterial strains (5×10^8^ CFU) daily. For NK cell depletion, mice were injected intraperitoneally with 200 µg of NK neutralizing antibody per dose. For bacterial OMV gavage experiments, tumor‐bearing mice were gavaged with 40 µg of OMVs every 2 days. For HK bacterial cells and bacterial culture supernatant gavage experiments, mice were gavage daily with heat‐inactivated bacteria (inactivated at 70°C for 10 min) at 5 × 10^8^ CFU and sterile culture supernatant (200 µL per dose). In the SP‐inhibited distant tumor model, tumor‐bearing mice were intraperitoneally injected with SP (Med Chem Express, HY‐101047, 10 mg/kg) every two days. For SP and checkpoint blockade synergy experiments, mice receiving immunotherapy were treated with 200 µg of anti‐PD‐1 (Bio X Cell, BE0146) per dose. For the experiment involving the combined treatment with SP and CCR5 antagonists, mice were treated with maraviroc (Med Chem Express, HY‐13004, 10 mg/kg) per dose. In the mouse CCL4 neutralization experiment, mice were intraperitoneally injected with SP (10 mg/kg) or DMSO every two days, and with CCL4 neutralizing antibody (Biolegend, 625504, 1 mg/kg) every three days. In the above animal models, 6 × 10^5^ LLC1 cells in 100 µl of PBS were injected subcutaneously into the flanks of mice. Mice were randomly divided into various treatment groups, with the treatment regimens detailed in the respective figures.

In the mild colitis model, percentage body weight loss was determined for each mouse by comparing its daily weight to the initial body weight before DSS administration. The disease activity index (DAI) was computed as the cumulative score of stool consistency, rectal bleeding, and percentage body weight loss. Stool consistency was graded as follows: 0, normal; 1, mildly soft; 2, very soft; 3, watery diarrhea. Rectal bleeding was scored as: 0, normal; 1, brown discoloration; 2, reddish discoloration; 3, gross blood. Body weight loss was scored according to the percentage reduction from baseline: 0 (<2%), 1 (2%–5%), 2 (5%–10%), 3 (10%–15%), 4 (≥15%). Daily scoring of body weight loss and DAI was performed throughout the DSS treatment period [58]. For histopathological evaluation, seven distinct parameters were assessed: (A) inflammation severity (0–4), (B) crypt damage extent (0–4), (C) neutrophil and lymphocyte infiltration (0–3), (D) submucosal edema (0–3), (E) goblet cell depletion (0–3), (F) reactive epithelial hyperplasia (0–3), and (G) crypt abscess formation (0–2) [58].

To detect the antitumor activity of SP‐stimulated mouse NK cells in vivo, splenic NK cells were purified by a mouse NK cell isolation kit (Biolegend, 480049), cultured in DMEM medium (Thermo Fisher Scientific, C11995500BT) containing 20% FBS, 1000 U/mL recombinant murine IL‐2 (Biolegend, 212‐12‐5) and 20 ng/mL recombinant murine IL‐15 (Biolegend, 210‐15‐10). After stimulation with 2 µm SP for 12 h, cells were mixed with LLC1 (6 × 10^5^ cells/mouse) at an E:T ratio of 1:1 and subcutaneously inoculated into the flanks of mice.

For xenograft A549 models, 2 × 10^6^ A549 cells in 100 µL of PBS were injected subcutaneously into the flanks of mice. NK‐92 cells stimulated with 5 µm SP for 12 h were intravenously injected into mice at E:T ratio of 1:1 every 4 days. Recombinant human IL‐2 (1 × 10^5^ U) was intraperitoneally injected every 4 days to support NK cell survival in vivo.

Tumor volume was assessed with a caliper and computed as (length × width^2^)/2.

### 4.9 Bacterial Identification

Bacterial genomic DNA was extracted using the Bacterial Genomic DNA Extraction Kit (TIANGEN, DP302‐02). To identify the bacterial species, primer pairs targeting the 16S rRNA gene were used to sequence the isolated strains: 27F (5’‐AGAGTTTGATCCTGGCTCAG‐3’) and 1492R (5’‐GGTTACCTTGTTACGACTT‐3’) at Sangon Biotech. The sequences obtained were matched against those in the EzBioCloud 16S rRNA databases (www.ezbiocloud.net).

### 4.10 16S rRNA Gene Sequencing Analysis

The Stool DNA Kit (Omega Bio‐tek, D4015‐02) was used to extract microbial genomic DNA from mouse feces samples according to the manufacturer's protocols. Absolute quantification of bacterial 16S rRNA amplicon sequencing was performed by Majorbio Bio‐Pharm Technology Co. Ltd., analyzed on the online platform of Majorbio Cloud Platform (https://cloud.majorbio.com/). The V3‐V4 hypervariable region of the 16S rRNA gene in bacteria was amplified using primer pairs 338F (5'‐ACTCCTACGGGAGGCAGCAG‐3') and 806R (5'‐GGACTACHVGGGTWTCTAAT‐3') by T100 Thermal Cycler PCR thermocycler (BIO‐RAD, USA). The PCR reaction mix consisted of 4 µL of 5 × Fast Pfu buffer, 2 µL of 2.5 mm dNTPs, 0.8 µL of each primer (5 µm), 0.4 µL of Fast Pfu polymerase, 10 ng of template DNA, and ddH_2_O to reach a total volume of 20 µL. The cycling conditions for PCR amplification are: The process starts with denaturation at 95°C for 3 min, followed by 27 cycles of 30‐second denaturation at 95°C, 30‐second annealing at 55°C, and 45‐second extension at 72°C, with a final extension at 72°C for 10 min, ending at 4°C.

Equimolar amounts of purified amplicons were combined and sequenced using paired‐end technology on an Illumina Nextseq2000 platform, following the standard protocols of Majorbio Bio‐Pharm Technology Co. Ltd. in Shanghai, China.

### 4.11 Single‐Cell Sequencing and Analysis

The tumor tissues were carried in a sterile culture dish containing 10 mL of 1x DPBS (Thermo Fisher, 14190144) on ice to eliminate any remaining tissue storage solution, and then chopped on ice. To digest the tissues, we utilized 0.25% trypsin (Thermo Fisher, 25200‐072) and 10 µg/mL DNase I (Sigma, 11284932001) dissolved in PBS containing 5% FBS. At 37°C and a shaking speed of 50 rpm, tumor tissues were dissociated for around 40 min. We gathered the dissociated cells every 20 min to boost cell yield and viability. Cell suspensions were passed through a 40 um nylon cell strainer, and red blood cells were removed with an ACK lysis buffer (Solarbio R1010). Dissociated cells were washed with 1x DPBS containing 2% FBS.

Essentially, 10x beads were subjected to second‐strand cDNA synthesis, adaptor ligation, and universal amplification. To enrich the 5’ end of transcripts connected with the cell barcode and UMI, sequencing libraries were prepared using randomly interrupted whole‐transcriptome amplification products. According to the manufacturer's instructions, single‐cell RNA libraries were prepared using the Chromium Single Cell 5’v2 Reagent and the Chromium Single Cell V(D)J Reagent kits, both from 10x Genomics. The sequencing libraries were measured with a High Sensitivity DNA Chip (Agilent) on a Bioanalyzer 2100 and the Qubit High Sensitivity DNA Assay (Thermo Fisher Scientific). Sequencing was performed on NovaSeq6000 (Illumina) using PE150 chemistry. With identical barcodes for each cell, we simultaneously examined immune repertoire (BCR/TCR) and gene expression from the same cell, allowing us to link clonotype to the corresponding cell subtype. The data analysis was conducted using the Majorbio Cloud Platform's online service (https://cloud.majorbio.com/).

### 4.12 Tissue Digestion and Single‐Cell Preparation

Tumor tissue was harvested and sliced into small pieces, followed by the addition of a digestion medium containing RPMI1640, 1 mg/mL collagenase IV (Sigma C5138), 40 mg/mL DNase I (Sigma 11284932001), and 2% heat‐inactivated FBS, and incubated for 40 min at 37°C. To obtain single‐cell suspensions, cells were filtered through a 70 mm cell strainer. Enrichment of hematopoietic cells was achieved by density gradient centrifugation with 30% Percoll (GE 17‐0891‐01) for 20 min at 1400 rpm at 4°C without breaks. The interphase containing hematopoietic cells was isolated and washed with PBS. ACK lysis buffer was used to lyse red blood cells.

### 4.13 Flow Cytometry

Cells were stimulated for 5 h at 37°C, 5% CO_2_ using a cell activation cocktail with Brefeldin A (Biolegend, 423303). For intracellular staining, cells were stained with the Zombie NIRTM Fixable Viability Kit (Biolegend, 423105). Cells were washed with PBS and stained with appropriate surface marker antibodies for CD4^+^ and CD8^+^ T cells and NK cells for 30 min at 4°C. Cells were fixed for 30 min with fixation buffer (Biolegend, 420801), followed by permeabilization using an intracellular staining permeabilization wash buffer (Biolegend, 421002). The intracellular antibody was added and incubated for 1 h at room temperature. The following antibodies were used for intracellular staining: anti‐mouse Perforin (Biolegend, 154305), anti‐mouse IFN‐γ (Biolegend, 505810), and anti‐mouse Gzmb (Biolegend, 372213). Cells were washed and characterized using an 18‐color BD LSR Fortessa (BD, Biosciences). Acquired data were analyzed using the FlowJo software.

### 4.14 Immunofluorescence Imaging

The tumor tissues, frozen in OCT (Sakura Finetek, 4583) compound, were sectioned into 5 µm slices. These slices were fixed in cold acetone for 10 min at 4°C and blocked with 5% bovine serum albumin (Sangon Biotech, A600332‐0100) for 1 h at room temperature. Samples were then incubated at 4°C overnight with the indicated primary antibodies: CD4 (Bioss, bs‐0766R, 1:250), CD8 (Bioss bs‐0648R, 1:250), Gzmb (Proteintech, 13588‐1‐AP, 1:250). After washing with PBS, the samples were incubated for an hour with a secondary antibody labeled with Alexa Fluor 488 (Proteintech, SA00013‐2, 1:400) or Alexa Fluor 594 (Proteintech, SA00013‐4, 1:400). DAPI (Solarbio, C0065) was used to stain the nuclei, and images were taken using a confocal fluorescence microscope (Leica TCS‐SP8, Leica Microsystems).

To observe the uptake of OMVs by NK‐92 cells in vitro, the outer membrane of OMVs was labeled with PKH26 (Solarbio, D0030). Briefly, OMVs (0.4 mg/mL) were suspended in PBS with PKH26 (1:250) and subsequently incubated at room temperature for 5 min. Termination was performed using FBS. Labeled OMVs (25 µg) were co‐incubated with NK‐92 cells for 30 min. After washing with PBS for two times, nuclei of NK‐92 cells were stained with DAPI for 10 min at room temperature. To observe the uptake of SP by NK‐92 cells in vitro, SP was labeled with 7ACC (Photo‐SP(7ACC)) by covalent bonding. Photo‐SP(7ACC) (5 µm) was co‐incubated with NK‐92 cells for 30 min. RedDot1 (Biotum, 40060‐T) was used to stain the nuclei. Cells were visualized and analyzed by a confocal fluorescence microscope (Leica TCS‐SP8, Leica Microsystems). The cells were observed and counted in three randomly chosen visual fields under microscope.

### 4.15 Fish

To evaluate the abundance of *B. intestinalis* in colon and tumor tissues after *B. intestinalis* gavage, fluorescence in situ hybridization (FISH) was performed on colon and tumor tissues using the DNA bacterial universal FISH Kit (D‐0016, Exonbio). 16S rRNA‐targeted oligonucleotide probes were synthesized by Focobio. The sequences of probes are listed in Supplementary Table S1. The images were captured with a confocal fluorescence microscope (Leica TCS‐SP8, Leica Microsystems).

### 4.16 Hematoxylin and Eosin (H&E) Staining

Histological analysis involved fixing the distal colon tissue with 4% paraformaldehyde, embedding it in paraffin, and slicing it into 5 mm sections. The slices were stained with hematoxylin and eosin, and images were captured using a microscope (BX46, Olympus).

### 4.17 LPS Concentration Measurement

Mouse peripheral blood serum was collected and measured for LPS levels according to the manufacturer's instructions (GenScript, L00350/L00350C). Plates were read using the Thermo Multiskan Ascent Microplate Reader (Thermo Scientific).

### 4.18 RNA Extraction and Real‐Time PCR

In the experiment to determine the effective components of bacteria, NK‐92 cells were treated with live bacteria (MOI = 200, 48 h), heat‐killed bacteria (MOI = 200, 48 h), bacterial culture supernatant (bacterial culture supernatant: cell culture medium = 1:1, 48 h), and bacterial culture supernatant fractions (bacterial culture supernatant: cell culture medium = 1:1, 48 h). In the transwell experiment, the bottom of the upper chamber of the transwell insert was coated with agar, and *B. intestinalis* (MOI = 200) was cultured in the upper chamber with medium added. NK‐92 cells were seeded in the lower chamber for 48 h of co‐cultivation.

To determine the effect of OMVs on NK cells, NK‐92 cells were treated with OMVs (50 µg/mL, 24 h) derived from *B. intestinalis* and *B. stercorirosoris*, or *B. i* OMVs (50 µg/mL, 24 h) cultivated under different pH values, temperatures, and H_2_O_2_ concentrations. To determine the types of active ingredients in *B. i* OMV, OMVs were subjected to 6 cycles of freeze–thawing, followed by 1 min of sonication and 30 s of cooling on ice to prepare the lysate. Subsequently, the lysate was treated with DNase I (2 U/L,) or proteinase K (40 µg/mL,) at 37°C for 30 min. An equal volume of the treated solution relative to the lysate was added to NK‐92 cells for a 24 h stimulation.

In the experiment of examining the effects of various metabolites, NK‐92 cells were treated with SP (Med Chem Express HY‐101047), Lyso‐PC (Med Chem Express, HY‐W251428), PG (Med Chem Express, HY‐W251428), PE (Sigma‐Aldrich, HY‐W250118), PC (Aladdin, L130333), PS (Med Chem Express, HY‐A0183), S1P (Med Chem Express, HY‐108496) at the concentrations indicated in the figure for 12 h.

To determine the effect of SP on CD8^+^ cells, CD8^+^ cells of the mouse spleen were treated with SP (5 µm, 12 h).

RNA from cells was extracted using the Trizol reagent (Solarbio) in accordance with the manufacturer's directions. Subsequently, cDNA synthesis was performed using a HiFiScript cDNA Synthesis Kit (CW2569M, Cwbio). qRT‐PCR was performed using SYBR Green qPCR Master mixes (CW0957M, Cwbio) on a thermocycler PCR system (QuantStudio 1, Applied Biosystems), and β‐actin was used as an endogenous control gene. 2^−ΔΔCT^ method was used to calculate the transcript levels of the indicated genes.

### 4.19 Preparation of Bacterial OMVs

Bacteroides were cultured at 37°C until they reached the logarithmic growth phase. After centrifugation at 6000 × g for 10 min at 4°C, the rotational speed was adjusted to 10 000 × g for another 30 min of centrifugation. The supernatant was collected and passed through a 0.22 µm sterile filter, followed by ultracentrifugation at 1 60 000 × g for 2 h at 4°C. The supernatant was discarded, and the OMVs were resuspended in 100 µl of PBS. After quick‐freezing in liquid nitrogen, the OMVs were stored in a ‐80°C refrigerator (Thermo).

### 4.20 Nanoparticle Tracking Analysis

Nanoparticle tracking analysis (NTA) was performed with NS300 nanoparticle analyzer (NanoSight, Malvern) to measure the size distribution of OMVs as described previously. We employed a camera level of 13–14 for every recording, with all post‐acquisition settings on automatic, except the detection threshold, which remained fixed at 5. To obtain a particle concentration ranging from 1 × 10^8^ to 1 × 10^9^ per milliliter, all samples were diluted in 0.22 µm filtered PBS at ratios between 1:100 and 1:1,000. The camera was focused to render the particles as sharp dots. Three 60‐second videos were recorded for each sample using the script control function, with a sample advance and a 5‐second delay between recordings.

### 4.21 Untargeted Metabolomics Analysis of Metabolites Derived From Bacterial OMVs

A 100 µL liquid sample was transferred into a 1.5 mL centrifuge tube containing 400 µL of an acetonitrile‐methanol mixture (1:1, v/v) supplemented with 0.02 mg/mL internal standard (L‐2‐chlorophenylalanine) for metabolite extraction. The mixture was incubated at −20°C for 30 min to facilitate protein precipitation. Subsequently, the samples were centrifuged at 13 000 × g and 4°C for 15 min. The resulting supernatant was carefully aspirated and dried under a stream of nitrogen. The dried residues were reconstituted in 100 µL of an acetonitrile‐water solution (1:1, v/v) and subjected to low‐temperature ultrasonication (5°C, 40 KHz) for 5 min for further extraction. After ultrasonication, the samples were centrifuged again at 13 000 × g and 4°C for 10 min, and the supernatant was transferred to autosampler vials for subsequent LC‐MS/MS analysis.

LC‐MS/MS analysis was performed using a Thermo UHPLC‐Q Exactive HF‐X system coupled with an ACQUITY HSS T3 column (100 mm × 2.1 mm i.d., 1.8 µm; Waters, USA) at Majorbio Bio‐Pharm Technology Co. Ltd. (Shanghai, China). The mobile phases comprised solvent A (0.1% formic acid in water‐acetonitrile, 95:5, v/v) and solvent B (0.1% formic acid in acetonitrile‐isopropanol‐water, 47.5:47.5, v/v). The flow rate was maintained at 0.40 mL/min, and the column temperature was set to 40°C. The full MS resolution was 60 000, and the MS/MS resolution was 7500. Data were acquired using the Data Dependent Acquisition (DDA) mode, with detection performed over a mass range of 70–1050 m/z.

Raw LC/MS data were preprocessed using Progenesis QI software (Waters Corporation, Milford), and a three‐dimensional data matrix in CSV format was generated and exported. The three‐dimensional matrix contained the following information: sample details, metabolite names, and mass spectral response intensities. Internal standard peaks, along with known false positive peaks (including noise, column bleed, and derivatized reagent peaks), were removed from the data matrix, followed by de‐redundancy and peak pooling.

### 4.22 SP Concentration Measurement

The OMVs of different species of Bacteroides and the *B. i* OMVs cultivated at two different temperatures were collected and measured for SP levels according to the manufacturer's instructions (Sbjbio, SBJ‐CR0054). Plates were read using the Thermo Multiskan Ascent Microplate Reader (Thermo Scientific).

### 4.23 NK‐92 Cell Viability Assay

Cell counting kit 8 (CCK8, Solarbio, CA1210) was used to measure the number of living NK‐92 cells. NK‐92 cells were treated with SP (5 µm) for 12 h. 10 µL of CCK8 solution was added onto a 96‐well plate seeded with NK‐92 cells and incubated for 4 h. Absorbance at 450 nm was recorded.

### 4.24 In Vivo OMV Trafficking Assays

PKH26‐labeled OMVs (40 µg per mouse) were delivered to mice through oral gavage. After 1, 5, 10, or 12 h of OMV gavage, peripheral blood and tumors were collected for detecting the appearance of PKH26 red fluorescence in NK cells by flow cytometry. After 12 h of OMVs gavage, colon tissues were collected for detecting the appearance of PKH26 red fluorescence in LP by a confocal fluorescence microscope (Leica TCS‐SP8, Leica Microsystems).

### 4.25 Co‐Culture of CD8^+^ T Cells With NK Cells

NK cells and CD8^+^ T cells were purified from mouse spleens using Mouse NK Cell Isolation Kit (Biolegend, 480050) and Mouse CD8^+^ Cell Isolation Kit (Biolegend, 480008). NK cells were pretreated with DMSO or 1 µM SP for 6 h, then co‐cultured with CD8^+^ T cells at a 1:1 ratio for 72 h. Post‐culture, cells were stained with anti‐CD8, anti‐IFN‐γ, and anti‐perforin antibodies, followed by flow cytometry to quantify IFN‐γ^+^ and perforin^+^CD8^+^ T cell frequency changes compared to untreated controls.

### 4.26 Mitochondrial Function Detection

Oxygen consumption rate was utilized to assess mitochondrial respiration with the Seahorse Bioscience Extracellular Flux Analyzer (XF96) (Seahorse Biosciences). Briefly, NK‐92 cells were plated onto XF Cell Culture Microplates and treated with DMSO or SP (5 µm) for 12 h. NK cells isolated from mouse spleens were directly plated into XF Cell Culture Microplates. Subsequently, the culture medium was replaced with XF assay medium (Agilent, 103576‐100), which consisted of XF base medium supplemented with 1 mm pyruvate (Seahouse Bioscience, 103578‐100), 2 mm GlutaMAX (Solarbio, G8180), and 2 mm glucose (Solarbio, G8150). The plate was then incubated in a CO_2_‐free incubator for 1 h for acclimatization before being loaded into the analyzer. A standard XF cellular mitochondrial stress test was performed, involving the sequential injection of oligomycin (2 µm, Med Chem Express, HY‐16589), FCCP (0.75 µm, Med Chem Express, HY‐100410), and a mixture of rotenone (5 µm, Med Chem Express, HY‐B1756) and antimycin A (5 µm, MKBio, MS0070).

Reactive oxygen species (ROS) levels in NK‐92 cells treated with DMSO or SP (5 µm) for 12 h and spleen NK cells of mice were measured using a Mitochondrial Superoxide Assay Kit (Beyotime, S0061S) following the manufacturer's protocols, with detection performed via flow cytometry (BD Biosciences). Briefly, the MitoSOX Red probe was diluted 1:1000 in serum‐free medium and incubated with the cells at 37°C for 20 min.

The mitochondrial membrane potential (MMP) change of NK‐92 cells treated with DMSO or SP (5 µm) for 12 h and spleen NK cells after treatment was detected with JC‐1 fluorescent probe according to the manufacturer's instruction via flow cytometry. NK‐92 cells and spleen NK cells of mice were incubated with JC‐1 working solution for 20 min at 37°C. Mitochondria with high membrane potential were labeled with red fluorescence (PE), whereas those with low membrane potential were labeled with green fluorescence (FITC). Mitochondrial membrane potential (MMP) was determined by the ratio of red to green fluorescence intensities.

### 4.27 Synthesis of Photoaffinity Probe Photo‐SP and Blue Fluorescent Label Probe Photo‐SP(7ACC)

The synthesis of 3‐(3‐(but‐3‐yn‐1‐yl)‐3H‐diazirin‐3‐yl)‐N‐((2S,3R,E)‐1,3‐dihydroxyoctadec‐4‐en‐2‐yl)propanamide (photo‐SP) were as follows. Accurately weigh 299.50 mg (1 mmol) of SP ((2S,3R,E)‐2‐aminooctadec‐4‐ene‐1,3‐diol) using an analytical balance and place it in a 25 mL round‐bottom flask. Add 10 mL of N, N‐dimethylformamide (DMF), 0.5 mL of DIPEA, 379.25 mg (1 mmol) of HBTU, and 263.25 mg (1 mmol) of 2,5‐dioxopyrrolidin‐1‐yl 3‐(3‐(but‐3‐yn‐1‐yl)‐3H‐diazirin‐3‐yl)propanoate in sequence. React at room temperature for 4 h under dark conditions. After confirming the completion of the reaction by TCL, pour the reaction solution into a 100 mL separatory funnel, add 100 mL of 1 mol/L hydrochloric acid, and extract three times with ethyl acetate. The resulting organic layer was washed with saturated sodium chloride solution, dried over anhydrous sodium sulfate, filtered, and concentrated. Photo‐SP (320.86 mg) was purified by HPLC with a yield of 71.68%.

The synthesis of 7‐(diethylamino)‐N‐(2‐(2‐(2‐(2‐(4‐(2‐(3‐(3‐(((2S,3R,E)‐1,3‐dihydroxyoctadec‐4‐en‐2‐yl)amino)‐3‐oxopropyl)‐3H‐diazirin‐3‐yl)ethyl)‐1H‐1,2,3‐triazol‐1‐yl)ethoxy)ethoxy)ethoxy)ethyl)‐2‐oxo‐2H‐chromene‐3‐carboxamide [photo‐SP(7ACC)] were as follows. To a solution of 7‐(diethylamino)‐2‐oxo‐2H‐chromene‐3‐carboxylic acid (7ACC) (261.27 mg, 1 mmol) in DCM (10 mL) were added DIPEA (0.6 mL), HBTU (379.25 mg, 1 mmol), and 2‐(2‐(2‐(2‐azidoethoxy)ethoxy)ethoxy)ethan‐1‐amine (218.26 mg, 1 mmol) and the reaction was stirred at room temperature under argon overnight. The reaction mixture was then concentrated and redissolved in ethyl acetate (30 mL). The organic layer was washed with saturated brine (20 mL). The organic layer was dried over anhydrous sodium sulfate, filtered, and concentrated. The residue was subjected to silica gel chromatography with methanol/dichloromethane (1:50) to 7ACC‐N3 (339.50 mg), a yellow oily substance, in 73.56% yield. Next, 7ACC‐N3 (23.76 mg, 0.05 mmol) and photo‐SP (22.38 mg, 0.05 mmol) were dissolved in 2.0 mL of DMF solution containing 10% water, and copper sulfate pentahydrate (12.50 mg, 0.05 mmol) and sodium ascorbate (10.00 mg, 0.05 mmol) were added, and the reaction was carried out at room temperature for 2 h. The mixture was filtered through a 0.22 µm filter membrane and purified using HPLC. Combine the organic phases collected by HPLC and concentrate to obtain 15.98 mg of yellow solid photo‐SP (7ACC) with a yield of 35.15%.

### 4.28 NK Cell Cytotoxicity Assay

To evaluate the effect of SP, NK‐92 cells were treated with DMSO or SP (5 µm) for 16 h. To investigate the role of oxidative phosphorylation, NK‐92 cells were pretreated with oligomycin (2 µm) for 2 h, followed by 16 h incubation with DMSO or SP (5 µm).

For in vitro cytotoxic assay, target K562 cells were labeled with CFSE (Biolegend 423801) according to the manufacturer's instructions. The treated effector cells were then co‐cultured with 3 × 10^4^ CFSE‐labeled K562 cells at various effector‐to‐target (E:T) ratios (5:1, 10:1, and 15:1) for 4 h in 96‐well U‐bottom plates. For in vivo cytotoxic assay, 2 × 10^6^ CFSE‐labeled A549 cells were mixed with the pre‐treated NK‐92 cells at an E:T ratio of 5:1. The cell suspension was then intraperitoneally injected into NSG mice. After 4 h, cells were harvested from the recipient mice by peritoneal lavage with cold PBS. After incubation, cells were stained with Propidium Iodide (PI, Med Chem Express HYD0815), and the percentage of dead target cells (CFSE^+^PI^+^) was quantified by flow cytometry.

### 4.29 Click Chemistry for Target Identification

For target profiling in cell lysate, NK‐92 cells were harvested, washed with pre‐chilled PBS, and lysed on ice for 30 min in lysis buffer supplemented with complete EDTA‐free Protease Inhibitor Cocktail (Roche, 11697498001). The lysate was sonicated and centrifuged at 20 000 × g for 20 min at 4°C to collect the soluble fraction. Protein concentration was determined by BCA assay and normalized to 2 mg/mL. For competitive profiling, lysates were pre‐incubated with the competitor SP (200 µm) or DMSO for 2 h, followed by the addition of the chemical probe DSP (200 µm) for another 2 h at 4°C. The mixtures were then placed on ice and irradiated with 365 nm UV light for 10 min. Following irradiation, the cross‐linked proteins were conjugated with Biotin‐PEG3‐Azide (Med Chem Express, HY‐130143) via Cu(I)‐catalyzed click chemistry reaction (1 mm CuSO_4_, 100 µm TBTA, 1 mm sodium ascorbate) for 2 h at room temperature.

Alternatively, for in situ labeling, NK‐92 cells (5 × 10^6^ cells/well) in serum‐free medium were pre‐treated with SP (10 µm) or DMSO for 2 h, followed by incubation with the chemical probe Pacsph (50 µm) or EtOH for an additional 2 h. After incubation, cells were washed twice with PBS and subsequently irradiated with 365 nm UV light for 30 min on ice. The cells were then lysed as described above. Protein concentrations of the lysates were quantified by BCA assay (Thermofisher Scientific, 23227). The normalized lysates were then subjected to a Cu(I)‐catalyzed click reaction with Biotin‐PEG3‐Azide under the same conditions as described above.

The reaction was quenched with ice‐cold methanol to precipitate proteins. The resulting pellet was washed twice with methanol, air‐dried, and resolubilized in 50 mm Tris‐HCl (pH 8.0) with 1% SDS, before being diluted to a final concentration of 0.1% SDS. The diluted protein solution was incubated with pre‐washed Streptavidin Agarose Beads overnight at 4°C with rotation. The beads were subsequently washed four times with PBS, followed by boiling in 1× SDS‐PAGE loading buffer for 10 min. The proteins were separated by SDS‐PAGE and visualized by silver staining (Thermofisher Scientific 24612) for subsequent LC‐MS/MS analysis.

For Western blot analysis, cell lysates were prepared as above, and a portion of the total cell lysate (input) was incubated with the following primary antibodies: anti‐ATP5F1A (1:5000, Proteintech, 14676‐1‐AP) and anti‐β‐actin (1:5000, ABclonal, AC004).

### 4.30 Western Blotting

NK‐92 cells were treated with SP (10 µm) or DMSO for 9 h. After treatment, total cell lysates were prepared using lysis buffer, and protein concentrations were normalized. The lysates were boiled at 99°C for 10 min and then separated by SDS‐PAGE before being transferred onto a polyvinylidene difluoride (PVDF) membrane. The membranes were blocked for 1 h at room temperature with 5% non‐fat milk in TBST and incubated overnight at 4°C with the following primary antibodies: anti‐ATP5F1A (1:5000, Proteintech, 14676‐1‐AP) and mouse anti‐β‐actin (1:5000, ABclonal, AC004). The next day, after washing with TBST, the membranes were incubated with HRP‐conjugated secondary antibodies for 1 h at room temperature. Protein bands were visualized using Immobilon Western Chemiluminescent HRP Substrate (Merck Millipore) and imaged on Amersham Imager 600 system (GE Healthcare Life Sciences). The band intensities were quantified using ImageJ software.

NK‐92 cells were treated with DMSO or SP in the presence or absence of CHX (TargetMol, T1225) for 9 h. NK‐92 cells were pretreated with MG132 (Solarbio, IM03101) for 2 h, followed by DMSO or SP treatment for 9 h. By using the above methods, the content of ATP5F1A protein was detected.

### 4.31 Measurement of ATP Synthase Activity

To assess enzymatic activity, NK‐92 cells were treated with SP (10 µm) or DMSO for 9 h. After treatment, the cells were harvested, and ATP synthase activity was measured using an ATP synthase enzyme activity microplate assay kit (Abcam, 109714) according to the manufacturer's instructions.

### 4.32 Measurement of Intracellular ATP Levels

Following a 9 h treatment with SP (10 µm) or DMSO, NK‐92 cells were harvested, and the ATP content was detected using the Enhanced ATP Assay Kit (Beyotime Biotechnology, S0027) according to the manufacturer's instructions.

### 4.33 Plasmid Construction and Lentivirus Production

A short hairpin RNA targeting human ATP5F1A (shATP5F1A; 5′‐CCGGCCTCTGTTGATCTTGAAGAAACTCGAGTTTCTTCAAGATCAACAGAGGTTTTTG‐3′) was cloned into the pLKO.1 vector. The pLKO.1‐Scramble shRNA vector served as a negative control. For lentivirus production, the resulting plasmid, along with packaging plasmids psPAX2 and the envelope plasmid pMD2.G, was co‐transfected into HEK293T cells using polyethylenimine (PEI, Polysciences, 23966). Viral supernatants were harvested at 48 and 72 h post‐transfection, filtered through a 0.45 µm syringe filter, and subsequently concentrated using PEG8000 precipitation.

### 4.34 Lentiviral Transduction of NK‐92 Cells

NK‐92 cells were seeded into a 6‐well plate and transduced with the concentrated lentiviral supernatant in the presence of 8 µg/mL Polybrene (Sigma‐Aldrich H9268). The plate was centrifuged at 700 × g for 1 h at 32°C (spinoculation). The transduced cells were subjected to selection with puromycin (1.5 µg/mL) to generate stable cell lines. Knockdown efficiency of ATP5F1A was subsequently confirmed by Western blot and/or qRT‐PCR analysis.

### 4.35 Surface Plasmon Resonance (SPR)

SPR experiments were performed with a Biacore T200 SPR system (Biacore, Cytiva). In brief, experiments were performed at 25°C in PBS‐P buffer with 5% DMSO. The recombinant protein ATP5F1A and ATP5F1A mutant protein were immobilized on CM5 chips by amine coupling, then the diluted ligand SP was flowed through the chips at 30 µL/min. The solvent correction was performed at the same time. Background binding to blank immobilized flow cells was subtracted, and affinity constant KD values were calculated using the 1:1 binding kinetics model built in the BIAcore T200 Evaluation Software (version 3.2).

### 4.36 Transmission Electron Microscopy

Negative staining with transmission electron microscopy (TEM) was used to observe OMVs as previously described [59]. In brief, isolated OMVs were placed on carbon‐formvar‐coated copper EM grids (Tianld) for 1 min, then the excess was removed with filter paper and negatively stained with a 2% uranyl acetate solution (BDH) in water for 1 min. Grids were air‐dried before analysis using a Tecnai G2 20 Twin TEM (FEI) at ×29 000 magnification.

### 4.37 Construction of the B. intestinalis^Δspt^ Mutant

The *B. intestinalis^Δspt^* mutant was generated using the CRISPR‐Cas system according to a previously reported method [60]. As *B. intestinalis* is intrinsically resistant to erythromycin, the erythromycin resistance gene (erm) on the pB025 vector was first replaced with a thiamphenicol resistance gene (catP) [61]. This new backbone vector, hereafter referred to as pB025‐CatP, was used for all subsequent cloning. A specific sgRNA (5'‐GTTATGTTTGTACTTCAATTGAGTA‐3') targeting spt was designed using CHOPCHOP (https://chopchop.cbu.uib.no/). Two homologous arms (HAs), each ∼1 kb, flanking the target gene were amplified from *B. intestinalis* genomic DNA. These HAs and the sgRNA cassette were then assembled into the linearized pB025‐CatP vector. The construct was transformed into *E. coli* S17‐1 λpir and verified by sequencing. The plasmid was subsequently transferred into *B. intestinalis* via conjugation at a 1:5 ratio (*E. coli* S17–1: *B. intestinalis*). After 24 h aerobic incubation at 37°C, the transconjugants were selected on gentamicin (200 µg/mL) and thiamphenicol (15 µg/mL) plates. Colonies confirmed by PCR to contain the constructed plasmid were incubated anaerobically in BHI medium with gentamicin (200 µg/mL) and thiamphenicol (25 µg/mL) overnight and diluted 1:100 into BHI medium containing gentamicin, thiamphenicol, and aTc (100 ng/mL) to induce the genome editing for 24 h. Then, cultures were plated on a BHI‐aTc plate and incubated anaerobically for 2 days. Potential mutants were screened by PCR, and candidates showing the correct gene deletion were confirmed by sequencing. Finally, the editing plasmid was cured from a confirmed mutant by serial passaging in an antibiotic‐free BHI plate. The final strain, designated *B. intestinalis^Δspt^*, was verified by its loss of antibiotic resistance. All primers used are listed in Table S1.

### 4.38 Molecular Docking

Molecular docking was performed to predict the potential binding interaction between sphingosine and human ATP synthase. The crystal structure of human ATP synthase was obtained from the Protein Data Bank (PDB ID: 8H9S). The 3D structure of sphingosine was retrieved from the PubChem database (CID: 5280335) and prepared for docking.

The docking analysis was conducted using the CB‐Dock2 web server (https://cadd.labshare.cn/cb‐dock2/), a platform that automatically identifies binding sites and performs blind docking using AutoDock Vina [62, 63, 64]. The simulation was run with default parameters. Following the docking procedure, the resulting protein‐ligand complex conformations were exported. The binding pose with the most favorable Vina score (representing the lowest binding free energy) was selected and visualized using PyMOL. The key amino acid residues involved in the interaction were then analyzed.

### 4.39 Datasets

Metagenomic data (PRJNA1023797, ERP134027) were analyzed by Iotabiome Biotechnology Co,. Ltd.

### 4.40 Statistical Analyses

Variance homogeneity was verified using the F‐test for comparisons involving two groups; for multi‐group analyses, either the Brown–Forsythe test or Bartlett's test was applied instead. For datasets conforming to the assumptions of normality and homoscedasticity, statistical significance between two independent groups was ascertained through the unpaired two‐tailed Student's t‐test, while one‐way or two‐way analysis of variance (ANOVA) was employed for multi‐group comparisons. In cases where data were normally distributed but exhibited heterogeneous variances, the unpaired two‐tailed Welch's t‐test was used for two‐group contrasts, and multi‐group analyses were performed via the Brown–Forsythe ANOVA or Welch's ANOVA. For non‐normally distributed data, the Mann–Whitney U test was selected to identify differences between two groups. A p‐value less than 0.05 was defined as statistically significant. All data were expressed as mean±standard error of the mean (SEM) and analyzed with GraphPad Prism software (GraphPad Software). Graphical Abstract were designed using BioRender (https://biorender.com/). All schematic illustrations were designed using Figdraw (www.figdraw.com).

## Author Contributions

Q.W. and Z.Z. designed the study and analyzed the data. K.Y., W.M., J.W., X.S., X.L., L.Y., and J.L. performed mouse experiments. J.Y. analyzed the scRNA‐seq data. K.Y., W.M., and X.P performed the extraction, identification of OMVs, and related experiments. Z.Y. assisted with the experimental design. X.S. and W.M. conducted the construction of *B. intestinalis ^ΔSPT^*, X.S., L.Z., Y.L., and T.R. performed the screening of SP target proteins, and related experiments on ATP synthase function.

## Funding

National Natural Science Foundation of China (NSFC) Programs (32570111, 82473963), CAMS Innovation Fund for Medical Sciences (CIFMS) (2025‐I2M‐3‐001), Science & Technology Development Fund of the Tianjin Education Commission for Higher Education (2023KJ039).

## Conflicts of Interest

Q. Wang, K. Yu, Z. Zhang, X. Sun, W. Ma, and X. Peng are co‐inventors on patent application related to sphingosine and lung cancer.

## Supporting information




**Supporting File**: advs75260‐sup‐0001‐SuppMat.docx

## Data Availability

The 16S rRNA‐seq and scRNA‐seq data generated in this study are available in the NCBI database under BioProject IDs PRJNA1313405 and PRJNA1295121. The untargeted metabolomics data generated in this study is available at the Metabolomics Workbench [65] (accession number PR002551). All other data that support the findings of this study are included in the present manuscript.
