# Supplementary material for: A Microbial Lipid‐ATP Synthase Axis Fuels NK Cell Antitumor Activity
Source: Adv Sci (Weinh). 2026 Apr 13:e20095. Online ahead of print. doi: 10.1002/advs.202520095 (PMC13334858; doi:10.1002/advs.202520095)
Supplement: Supplementary file 1 [file ADVS-9999-e20095-s001.docx]

Supporting Information 

**A Microbial Lipid-ATP Synthase Axis Fuels NK Cell Antitumor Activity**

*Kaiyuan Yu, Xinyu Sun, Wanxia Ma, Jianming Yang, Xuan Sun, Lisong Zhang, Yumeng Liu, Tianshu Ren, Qi Wang, Jingyu Wang, Xiao Li, Xianping Peng, Liu Yang, Junqiang Lv, Zhi Yao, Zhi-Song Zhang*, Quan Wang** 

**Supplementary Figure 1. Mild colitis-associated gut microbiota inhibit extra-intestinal tumor growth.**

**A-E**, Schematic of DSS treatment and LLC1 tumor progression. Mice received 1% DSS or water for 8 days, with LLC1 inoculated on day 0 (**A**). Tumor growth curves (**B**) and tumor weights (**C**) in DSS- and water-treated groups after LLC1 implantation (*n* = 9 mice/group). Tumor growth curves (**D**) and tumor weights (**E**) after B16 cell implantation in DSS- or water-treated groups (*n* = 4 mice/group). **F-J**, Schematic of DSS treatment. Mice received DSS or water for 8 days (**F**). Analysis of disease activity index (**G**), colon length (**H**), histopathological score (**I**) and mRNA expression levels of inflammatory cytokines in the colon (**J**) post DSS administration for the water, 1%DSS group, and 3%DSS group. **K-N**, Schematic of ABX treatment and LLC1 tumor progression (**K**). Bacterial DNA levels in ABX- or ABX+DSS-treated groups (**L**) (*n* = 6 mice/group). ,Tumor growth curves (M) and tumor weights (**N**) after LLC1 implantation in ABX- or ABX+DSS-treated groups (*n* = 6 mice/group). **O**,**P**, Tumor growth curves (**O**) and tumor weights (**P**) of LLC1 tumors in *Flavonifractor plautii* (*F. p*) and *RIAY harlan* (*R. h*) groups, from the model in Figure 1E (*n* = 4 mice/group). **Q**,**R**, Tumor growth curves (**Q**) and tumor weights (**R**) of LLC1 tumors in *Bacteroides acidifaciens* (*B. a*) and *Bacteroides thetaiotaomicron* (*B. t*) groups, from the model in Figure 1E (*n* = 4 mice/group). **S,T**, Gating strategies for flow cytometry analysis in tumor and peripheral blood (PB) (**S**) and in lamina propria (**T**). All data are presented as mean ± s.e.m. Statistical analysis: two-way ANOVA followed by Sidak’s comparison test (**B**,**D**,**G**,**M**,**O,Q**), two-tailed Mann-Whitney test (**C**,**E**,**N**) or one-way ANOVA followed by Tukey’s multiple comparisons test or Tukey's multiple comparisons test (**H-J,P,R**).

**Supplementary Figure** 2. **B. intestinalis** promotes the number and function of NK cells and CD8⁺ T cells in tumors.
A, tSNE plot of scRNA-seq clusters of NK cells from the two groups in the model of Fig. 1E. B, Heatmap of distinct expression patterns across NK cell subclusters. The top 10 most highly expressed genes per subcluster are shown. C, Volcano plots of differentially expressed genes in CD8⁺ T cells between the two groups. D, KEGG pathway enrichment analysis of upregulated genes in CD8⁺ T cells from the B. intestinalis group. E, tSNE plot of scRNA-seq clusters of CD8⁺ T cells. F, Heatmap of distinct expression patterns across CD8⁺ T cell subclusters. The top 10 most highly expressed genes per subcluster are shown. G, Proportions of CD8⁺ T cell subclusters in the two groups. H, Dot plots of distinct CD8⁺ T cell phenotype signature gene expression across subclusters. I, Volcano plots of differentially expressed genes in CD8⁺ T cell cluster 0 between the two groups. J–N, Proportions and counts of CD4⁺ T, CD8⁺ T, and NK cells in LLC1 tumors, peripheral blood (PB) or colonic lamina propria (LP) from the model in Fig. 1E (n = 6 mice/group). O–Q, Immunofluorescence analysis of Gzmb⁺ NK, CD8⁺, and CD4⁺ T cells in tumors from the model in Fig. 1E. Scale bar: 10 μm (n = 5 mice/group). R, Immunofluorescence analysis of NK cells in tumors from the model in Figure 2P. Scale bar: 10 μm (n = 5 mice/group). All data are presented as mean ± s.e.m. Statistical analysis: one-way ANOVA followed by Tukey’s multiple comparisons test (J–N, R) or two-tailed Mann-Whitney test (O–Q) .

**Supplementary Figure 3**. **B. intestinalis** does not colonize in extra-intestinal tumors.
A, Colonization of B. intestinalis in mouse colons was detected by FISH using the B. intestinalis probe, EUB338 probe (targeting eubacteria 16S rRNA) and the NON338 negative probe (Scale bar: 10 μm). B, Colon length of mice gavaged with PBS, B. intestinalis (B. i), or P. clara (P. c) . **C**,D, HE staining of colon tissues and LPS levels in peripheral blood of mice gavaged with PBS or B. intestinalis. **E**,F, *B. intestinalis* in the tumor of mice after gavage was detected by PCR (**E**) or FISH using the B. intestinalis probe (**F**) (Scale bar: 10 μm). G-I, Schematic of LLC1 tumor inoculation with heat-killed B. intestinalis gavage (G). Tumor growth curves (**H**) and tumor weights (**I**) in mice treated as indicated (*n* = 5 mice/group). **J-L**, Schematic of LLC1 tumor inoculation with B. intestinalis supernatant gavage (**J**). Tumor growth curves (**K**) and tumor weights (**L**) in mice treated as indicated (*n* = 5 mice/group). All data are presented as mean ± s.e.m. Statistical analysis: two-tailed Mann-Whitney test (**B**,D) , one-way ANOVA followed by Tukey’s multiple comparisons test (**I**,**L**),or two-way ANOVA followed by Sidak’s comparison test (**H**,**K**).

**Supplementary Figure 4**. **B. intestinalis**-derived OMVs cross the intestinal barrier, accumulate in blood NK cells and reach subcutaneous tumors.
A,B, NK-92 cells were stimulated with heat-killed B. intestinalis (MOI = 200, 48 h) (A) or culture supernatant (500 μL, <3 kDa or >3 kDa, 24 h) (B), followed by qRT-PCR analysis of Ifng mRNA levels (n = 4). C,D, Nanoparticle tracking analysis (NTA) (C) and representative TEM images (D) of B. stercorirosoris-derived OMVs (Scale bar: 200 nm). E, Localization and uptake of B. intestinalis-derived OMVs by NK-92 cells. OMVs labeled by PKH26 were co-incubated with NK-92 cells for 20 minutes and observed under a fluorescence microscope. **F-I**, Schematic of in vivo OMV tracking (F), uptake of PKH26-labeled B. intestinalis OMVs by peripheral blood NK cells at different time points after oral administration (G) and intratumoral NK cells (**I**) detected by flow cytometry, and fluorescence microscopy of colon cryosections showing PKH26-labeled OMVs (Scale bar: 10 μm) (**H**). J-M, Proportions of CD4⁺ T, total CD8⁺ T, IFN-γ⁺, and CD107a⁺ CD8⁺ T cells in tumors from mice in the model of Figure 3G (n = 5 mice/group). All data are presented as mean ± s.e.m. Statistical analysis: two-tailed Mann-Whitney test (A) or one-way ANOVA followed by Dunnett's multiple comparisons test (B, J–M).

**Supplementary Figure 5**. The impact of metabolites elevated in **B. intestinalis**-derived OMVs on NK cells.
A-C, NK-92 cells were stimulated with B. intestinalis (34 °C) OMVs cultured at different pH values (A) or varying H₂O₂ concentrations (B), followed by qRT-PCR analysis of Ifng mRNA levels (n = 4). OMV lysates were treated with DNase (2 U/L) or proteinase K (40 μg/mL) for 30 minutes and used to stimulate NK-92 cells for 24 hours and Ifng mRNA levels were analyzed (C). D-G, Principal component analysis (PCA) of metabolites from 34 °C OMVs, 43 °C OMVs, or GAM broth (D). Volcano plots of differentially expressed metabolites between 34  °C and 43  °C OMVs (E) . KEGG pathway enrichment analysis of upregulated metabolites in the 34 °C OMV group (F) . Venn diagram showing overlapping metabolites upregulated in the 34 °C OMV group compared to 43 °C and GAM groups (G) (n = 3). H-M, NK-92 cells were stimulated with phosphatidylglycerol (PG), phosphatidylethanolamine (PE), phosphatidylcholine (PC), lysophosphatidylcholine (Lyso-PC), phosphatidylserine (PS), or sphingosine 1-phosphate (S1P) for 12 hours, followed by qRT-PCR analysis of Ccl4, Gzmb, Ifng mRNA levels (n = 4). N, Metabolite levels of SP in B. intestinalis OMVs cultured at 34  °C or 43  °C. **O-P** Schematic illustration for investigating the uptake of SP by NK-92 cells (O). Fluorescence microscopy imaging shows that Photo-SP(7ACC) enters the NK-92 cells. (Scale bar: 10 μm) (**P**). NK-92 cell viability measured by CCK8 assay after 12-hour treatment with DMSO or SP (5 μm) (n = 4) (**Q**). CD8⁺ T cells were stimulated with DMSO or SP (5 μm) for 12 hours, followed by qRT-PCR analysis of Ifng, Gzmb, Ccl5, Gzma, and Prf1 mRNA levels (n = 4) (**R**) . **S**, NK cells isolated from mouse spleens were pretreated with DMSO or 1 μM SP for 6 h, then co-cultured with splenic CD8^+^ T cells at a 1:1 ratio for 72 h. The proportions of IFN-γ^+^ and perforin^+^ CD8^+^ T cells were subsequently detected by flow cytometry (n = 4). All data are presented as mean ± s.e.m. Statistical analysis: one-way ANOVA followed by Dunnett's multiple comparisons test or Sidak's multiple comparisons test (A-B, H-M, S) or two-tailed Mann-Whitney test (**C**, **N**, **Q-R**)

**Supplementary Figure 6**. Effects of sphingosine in **B. intestinalis**-derived OMVs on certain immune cells in vivo.
**A**. Mice were intraperitoneally injected with SP (10 mg/kg), and plasma samples were collected at 1 and 6 h post-injection for the measurement of SP levels. B-E, Proportions and counts of CD4⁺ T, total CD8⁺ T, IFN-γ⁺, and CD107a⁺ CD8⁺ T cells in tumors from the DMSO- or SP-treated mice in the model of Fig. 4A (n = 6 mice/group). F, scRNA-seq analysis of Ccl4 and Ccr5 expression in NK cells and NK-Cluster 0 in the PBS- or B. intestinalis-treated mice in the model of Fig. 1E. G, mRNA levels of Ccr5 in NK-92 cells stimulated with SP (12 h) were analyzed by qRT-PCR (n = 4). **H**, CCL4 levels in the tumors from the four groups in the model of Figure 4I (n = 5 mice/group). I, Proportions of total NK cells from tumors obtained from mice in the model of Figure 4J. (n = 5 mice/group) **J-N**, Mice received water, 1% or 3% DSS for 8 days and LLC1 cells were inoculated on day 0 (**J**). Tumor growth curves (**K**) and tumor weights (**L**) in water, 1% DSS- or 3% DSS-treated groups (n = 6 mice/group). **M**, Proportions of IFN-γ^+ ^NK cells in tumors. **N**, Plasma SP levels in mice treated with water and different concentrations of DSS. O, Schematic diagram for constructing *B. i*^*Δspt*^ mutant using CRISPR-Cas12a-mediated genome editing. **P,** PCR identification of of *B. i*^*Δspt*^ mutant. **Q**–T, Proportions of total NK, CD4⁺ T, total CD8⁺ T, IFN-γ⁺, and CD107a⁺ CD8⁺ T cells in peripheral blood (Q–R) and tumors (S–T) from the mice treated with B. i OMVs and *B. i*^*ΔSPT*^ OMVs in Figure S7J (n = 5 mice/group). All data are presented as mean ± s.e.m. Statistical analysis: two-tailed unpaired Student’s t test (A), two-tailed Mann-Whitney test (B–E, G), one-way ANOVA followed by Dunnett's multiple comparisons test or Sidak's multiple comparisons test (**H-I**, **L**–N, Q-T) or two-way ANOVA followed by Sidak’s comparison test (K).

**Supplementary Figure 7**. Sphingosine enhances tumor sensitivity to immune checkpoint therapy.
**A-E**, Schematic of tumor-suppressive model with SP and anti-PD-1 treatment. Mice received intraperitoneal injections of SP (10 mg/kg) or DMSO every other day and anti-PD1 (200 µg) every three days after tumor implantation (**A**). Tumor growth curves (B) and tumor weights (C) in four treatment groups of LLC1 tumors in mice. Proportions of total CD8⁺ and IFN-γ⁺ CD8⁺ T cells in LLC1 tumors (D–E) (n = 6 mice/group). **F**, *B. intestinalis* abundance of available metagenomic data from three tumor clinic cohorts: PRJNA1195622 [n = 29 (NR: non-responder) or 30 (R: responder)], PRJNA1023797 [n = 140 (NR) or 105 (R)] and ERP134027 [n = 51 (NR) or 55 (R)]. **G**, Serum SP levels in available metabolomic datasets data from one tumor clinic cohort: n = 76 (HC: healthy control) or 113 (CRC: colorectal cancer)]. **H**, SP levels in OMVs derived from B. intestinalis and B. intestinalis *ΔSPT* mutant (*B. i*^*ΔSPT*^) (n = 4). **I**, NK-92 were stimulated with *B. i* OMVs and *B. i*^*ΔSPT*^ OMVs (50 ug/ml, 24 h), followed by analysis of mRNA levels of *Ifng* by qRT-PCR (*n* = 4). J-M, Tumor outcomes in the model of Figure 3G. Tumor growth curves (**J**) and tumor weights (**K**) of LLC1 tumors in mice treated with OMVs. Proportions of NK and IFN-γ⁺ NK cells in peripheral blood (**L**) and tumors (**M**) (n = 5 mice/group). **N,** SPR analysis of mutant ATP5F1A–SP interaction. SP was passed at serial dilutions (45–10.68 µM) to immobilized triple-mutant ATP5F1A (G207A/A236V/Q243A) under identical conditions as in (Figure 5H). RU, response units. **O**, RT-qPCR analysis of *Atp5f1a* mRNA levels in NK-92 cells treated with DMSO or SP for 9 h (*n* = 4). **P**, Western blotting analysis of ATP5F1A in NK-92 cells treated with DMSO or SP in the presence or absence of CHX for 9h (*n* = 3). **Q**, Western blotting analysis of analysis of ATP5F1A in NK-92 cells pretreated with MG132 for 2 h, followed by DMSO or SP treatment for 9 h (*n* = 3). **R**, *Atp5f1a* mRNA levels of scramble or ATP5F1A *s*hRNA*-*treated NK-92 cells were analyzed (*n* = 4). All data are presented as mean ± s.e.m. Statistical analysis: two-way ANOVA followed by Sidak’s comparison test (B, **J**) , one-way ANOVA followed by Dunnett's multiple comparisons test or Sidak's multiple comparisons test (**C-E**, **I**, **K**–**M, P-Q**) or two-tailed Mann-Whitney test (**F-H,** **O, R**).

**Supplementary Table 1**

| **REAGENT or RESOURCE** | **SOURCE** | **IDENTIFIER** |
| --- | --- | --- |
| **Antibodies** |  |  |
| Anti-mouse PD-1 | Bio X Cell | Cat# BP0146 RRID: 10949053 |
| Anti-mouse Perforin-PE | Biolegend | Cat# 154305  RRID: AB_2721638 |
| Anti-mouse IFN-γ-APC | Biolegend | Cat# 505810 RRID: AB_315104 |
| Anti-mouse Gzmb-PEcy7 | Biolegend | Cat# 372213 RRID: AB_2728380 |
| Anti-mouse CD4 antibody | Bioss | Cat# bs-0766R RRID: AB_10857931 |
| Anti-mouse CD8 antibody | Bioss | Cat# bs-0648R RRID: AB_10857537 |
| Anti-mouse Gzmb antibody | Proteintech | Cat# 13588-1-AP RRID: AB_2114429 |
| Alexa Fluor 594 - labeled second antibody | Proteintech | Cat# SA00013-4 RRID: AB_2810984 |
| Alexa Fluor 488 - labeled second antibody | Proteintech | Cat# SA00013-2 RRID: AB_2797132 |
| Anti-mouse CD107a-FITC | Biolegend | Cat# 121605 RRID: AB_572006 |
| Anti-mouse CD45-eFluor 506 | Invitrogen | Cat# 69-0451-82 RRID: AB_2637147 |
| Anti-mouse CD3-BV421 | Biolegend | Cat# 100227 RRID: AB_10900227 |
| Anti-mouse CD4-FITC | Biolegend | Cat# 100406 RRID: AB_312691 |
| Anti-mouse CD8a-Percpcy5.5 | Biolegend | Cat# 100734 RRID: AB_2075238 |
| Anti-mouse NK1.1-Percpcy5.5 | Biolegend | Cat# 108728 RRID: AB_2132705 |
| Anti-mouse CD3ε | Biolegend | Cat# 100340 RRID: AB_11149115 |
| Anti-mouse CD28 | Biolegend | Cat# 122021 RRID: AB_2810370 |
| Anti-human IFN-γ-PE | Biolegend | Cat# 383303 RRID: AB_2924583 |
| β-Actin antibody | ABclonal | Cat# AC004 RRID: AB_2737399 |
| ATP5F1A antibody | Proteintech | Cat# 14676-1-AP RRID: AB_2061761 |
| Anti-Mouse IgG | SeraCare | Cat# 5220-0341 RRID: AB_2891080 |
| Anti-Rabbit IgG | SeraCare | Cat# 5220-0336 RRID: AB_2857917 |
| Anti-Mouse CCL4 neutralizing antibody | Biolegend | Cat# 625504 RRID: AB_2814489 |
| **Bacterial Strains and plasmid** |  |  |
| *Bacteroides intestinalis* | This paper | N/A |
| *Bacteroides acidifaciens* | This paper | N/A |
| *Bacteroides thetaiotaomicron* | This paper | N/A |
| *Flavonifractor plautii* | This paper | N/A |
| *Paraprevotella clara* | This paper | N/A |
| *RIAY harlan* | This paper | N/A |
| *Flavonifractor plautii* | This paper | N/A |
| *Bacteroides fragilis* | This paper | N/A |
| *Bacteroides koreensis* | This paper | N/A |
| *Bacteroides oleiciplenus* | This paper | N/A |
| *Bacteroides stercoris* | This paper | N/A |
| *Bacteroides thetaiotaomicron* | This paper | N/A |
| *E.coli S17-1* competent cells | Beyotime | Cat# D1075S |
| *E. coli* DH5α (DE3) | Solarbio | Cat# C1100 |
| pB025 | From Prof. Dai lab | N/A |
| pB025-CatP | This paper | N/A |
| pB025-CatP-Δ*spt* | This paper | N/A |
| pLKO.1 | Lab stored | N/A |
| pLKO.1-scramble-shRNA | Lab stored | N/A |
| pLKO.1-shATP5F1A | This paper | N/A |
| psPAX2 | Lab stored | N/A |
| pMD2.G | Lab stored | N/A |
| pMTL82151 | Miaolingbio | Cat# P29888 |
| pET28a-ATP5F1A-tri-mutant | This paper | N/A |
| **Cell lines** |  |  |
| NK-92 cell lines | ATCC | Cat# CRL-2407 |
| K562 cell lines | ATCC | Cat# CCL-243 |
| A549 cell lines | ATCC | Cat# CCL-185 |
| PK136 cell lines | ATCC | Cat# HB-191 |
| HEK293T cell lines | ATCC | Cat# CRL-3216 |
| **Chemicals, peptides, and recombinant proteins** |  |  |
| Fetal bovine serum | Gibco | Cat# A5256701 |
| PBS | Life-iLab | Cat# AC08L011 |
| Glycerine | Solarbio | Cat# G8190 |
| Pristane | Solarbio | Cat# T7330 |
| DPBS | Thermo Fisher | Cat# 14190144 |
| DAPI | Solarbio | Cat# C0065 |
| Reddot1 | Biotum | Cat# 40060-T |
| PKH26 | Solarbio | Cat# D0030 |
| Proteinase K | CWbio | Cat# CW2584M |
| Phosphatidylglycerol | Med Chem Express | Cat# HY-W251428 |
| Phosphatidylethanolamine | Med Chem Express | Cat# HY-W250118 |
| Phosphatidylcholine | Aladdin | Cat# L130333 |
| Lysophosphatidylcholine | Med Chem Express | Cat# HY-W251428 |
| Phosphatidylserine | Med Chem Express | Cat# HY-A0183 |
| Sphingosine-1-phosphate | Med Chem Express | Cat# HY-108496 |
| Sphingosine | Med Chem Express | Cat# HY-101047 |
| Maraviroc | Med Chem Express | Cat# HY-13004 |
| RNA Extraction Kit | Solarbio | Cat# R1200 |
| XF base medium | Agilent | Cat# 103576-100 |
| Pyruvate | Seahouse Bioscience | Cat# 103578-100 |
| GlutaMAX | Solarbio | Cat# G8180 |
| Glucose | Solarbio | Cat# G8150 |
| Oligomycin | Med Chem Express | Cat# HY-16589 |
| FCCP | Med Chem Express | Cat# HY-100410 |
| Rotenone | Med Chem Express | Cat# HY-B1756 |
| Antimycin | MKBio | Cat# MS0070 |
| Intracellular staining permeabilization wash buffer | Biolegend | Cat# 421002 |
| GAM broth | Hope Bio-Technology | Cat# HB8518 |
| Vitamin K1 | Hope Bio-Technology | Cat# HB0310b |
| Hemin | Hope Bio-Technology | Cat# HB0310a |
| Sheep blood | Hope Bio-Technology | Cat# 1001339-1 |
| Agar | Solarbio | Cat# A8190 |
| Yeast extract | OXOID | Cat# LP0021B |
| Typtone | OXOID | Cat# LP0042B |
| Sodium Chloride | Biosharp | Cat# BS112 |
| α-MEM medium | Thermo Fisher Scientific | Cat# C12571500BT |
| Inositol | Sigma | Cat# I7508 |
| β-mercaptoethanol | Thermo Fisher Scientific | Cat# 21985023 |
| House serum | Pricella | Cat# 164215 |
| Folic acid | Sigma | Cat# F7876 |
| Recombinant human IL-2 | Peprotech | Cat# 96-200-02-50 |
| RPMI1640 medium | BasalMedia | Cat# L220KJ |
| DMEM medium | Thermo Fisher Scientific | Cat# C11995500BT |
| HEPES | Sigma | Cat# 83264 |
| Non-essential amino acids  (NEAA) | Gibco | Cat# 11140-050 |
| Penicillin-streptomycin | Life-iLab | Cat# AC03L332 |
| Recombinant murine IL-15 | Peprotech | Cat# 210-15-10 |
| Ampicillin | SangonBiotech | Cat# A610028-0025 |
| Neomycin | SangonBiotech | Cat# A610366-0025 |
| Metronidazole | SangonBiotech | Cat# A600633-0025 |
| Vancomycin | SangonBiotech | Cat# A600983-0001 |
| Brain Heart Infusion | Hopebio | Cat# HB8297-5 |
| L-cysteine | Macklin | Cat# L804954 |
| Dextran Sodium Sulfate | MP Biomedicals | Cat# 0216011090 |
| Fixation buffer | Biolegend | Cat# 420801 |
| Recombinant murine IL-2 | Peprotech | Cat# 212-12-20 |
| DPBS | Thermo Fisher | Cat# 14190144 |
| Trypsin | Thermo Fisher | Cat# 25200-072 |
| DNase Ⅰ | Sigma | Cat# 11284932001 |
| ACK lysis buffer | Solarbio | Cat# R1010 |
| Collagenase IV | Sigma | Cat# C5138 |
| Percoll | GE | Cat# 17-0891-01 |
| Cell activation cocktail with Brefeldin A | Biolegend | Cat# 423303 |
| OCT | Sakura Finetek | Cat# 4583 |
| Erythromycin | Sigma-Aldrich | Cat# E0774 |
| Gentamicin | Sangon Biotech | Cat# A506614-0005 |
| Thiamphenicol | Solarbio | Cat# T9680 |
| PrimeSTAR® Max DNA Polymerase | Takara | Cat# R045A |
| Q5 High-Fidelity DNA Polymerase | New England Biolabs | Cat# M0491V |
| Anhydrotetracycline hydrochloride | Med Chem Express | Cat# HY-118660 |
| Propidium Iodide | Med Chem Express | Cat# HY-D0815 |
| Complete mini-EDTA-free protease inhibitor cocktail tablets | Roche | Cat# 11697498001 |
| Biotin-PEG3-Azide | Med Chem Express | Cat# HY-130143 |
| Cupric sulfate | Aladdin | Cat# C573445 |
| TBTA | Aladdin | Cat# T162437 |
| Sodium ascorbate | Aladdin | Cat# S105024 |
| Streptavidin Agarose Beads | Yeasen | Cat# 20512ES08 |
| DMSO | Solarbio | Cat# D8372 |
| Polybrene | Sigma-Aldrich | Cat# H9268 |
| Polyethylenimine | Polysciences | Cat# 23966 |
| Puromycin | Solarbio | Cat# P8230 |
| Recombinant protein ATP5F1A | Cusabio | Cat# CSB-EP002344HU |
| Cycloheximide | TargetMol | Cat# T1225 |
| MG132 | Solarbio | Cat# IM03101 |
| **Animal** |  |  |
| C57BL/6J mice | SPF Biotechnology Co., Ltd. | N/A |
| NOD-Cg-*Prkdc*^*scid*^*Il2rg*^*em1cya*^/Cya (NSG) mice | Cyagen | N/A |
| Balb/c mice | SPF Biotechnology Co., Ltd. | N/A |
| **Critical Commercial assays** |  |  |
| Mouse NK cell isolation kit | Biolegend | Cat# 480049 |
| Mouse CD8^+^ T cell isolation kit | Biolegend | Cat# 480008 |
| DNA bacterial universal FISH Kit | Exonbio | Cat# D-0016 |
| Zombie NIRTM Fixable Viability Kit | Biolegend | Cat# 423105 |
| LPS assay kit | GenScript | Cat# L00350/L00350C |
| Bacterial Genomic DNA Extraction Kit | TIANGEN | Cat# DP302-02 |
| Stool DNA Kit | Omega Bio-tek | Cat# D4015-02 |
| HiFiScript cDNA Synthesis Kit | Cwbio | Cat# CW2569M |
| SYBR Green qPCR Master mixes | CW0957M | Cat# Cwbio |
| Mitochondrial Superoxide Assay Kit | Beyotime | Cat# S0061S |
| Mitochondrial membrane potential assay kit with JC-1 | Beyotime | Cat# C2005 |
| Sphingosine assay kit | Sbjbio | Cat# SBJ-CR0054 |
| Cell counting kit 8 | Solarbio | Cat# CA1210 |
| Total RNA Extraction Kit | Solarbio | Cat# R1200 |
| ClonExpress Ultra One Step Cloning kit V3 | Vazyme | Cat# C117-01 |
| FastPure Gel DNA Extraction Mini Kit | Vazyme | Cat# DC301 |
| CFSE Cell Division Tracker Kit | Biolegend | Cat# 423801 |
| Pierce™ BCA Protein Assay Kits | Thermofisher Scientific | Cat# 23227 |
| Pierce™ Silver Stain Kit | Thermofisher Scientific | Cat# 24612 |
| Immobilon®-PSQ PVDF Membrane | Merck | Cat# ISEQ00010 |
| Immobilon Western Chemiluminescent HRP Substrate | Merck | Cat# WBKLS0500 |
| Enhanced ATP Assay Kit | Beyotime | Cat# S0027 |
| ATP synthase Enzyme Activity Microplate Assay | Abcam | Cat# 109714 |
| **Oligonucleotides** |  |  |
| Human-*Ccl4* F: CTGTGCTGATCCCAGTGAATC | Sangon Biotech | Custom made |
| Human-*Ccl4* R: TCAGTTCAGTTCCAGGTCATACA | Sangon Biotech | Custom made |
| Human-*Gapdh* F: ACGGATTTGGTCGTATTGGG | Sangon Biotech | Custom made |
| Human-*Gapdh* R: TGATTTTGGAGGGATCTCGC | Sangon Biotech | Custom made |
| Human-*Ifng* F: TCGGTAACTGACTTGAATGTCCA | Sangon Biotech | Custom made |
| Human-*Ifng* R: TCGCTTCCCTGTTTTAGCTGC | Sangon Biotech | Custom made |
| Human-*Gzmb* F: CCCTGGGAAAACACTCACACA | Sangon Biotech | Custom made |
| Human-*Gzmb* R: GCACAACTCAATGGTACTGTCG | Sangon Biotech | Custom made |
| Human-*Ccl3*                       F: GCTCTCTGCAACCAGTTCTC | Sangon Biotech | Custom made |
| Human-*Ccl3* R: GGCTTCGCTTGGTTAGGAAG | Sangon Biotech | Custom made |
| Mouse-*β-Actin* F: CACTGTCGAGTCGCGTCCA | Sangon Biotech | Custom made |
| Mouse-*β-Actin* R: GACCCATTCCCACCATCACA | Sangon Biotech | Custom made |
| Mouse-*Il-6* F: AGACAAAGCCAGAGTCCTTCAG | Sangon Biotech | Custom made |
| Mouse-*Il-6* R: GAGCATTGGAAATTGGGGTAGG | Sangon Biotech | Custom made |
| Mouse-*Il-1β* F: GGGCTGGACTGTTTCTAATGC | Sangon Biotech | Custom made |
| Mouse-*Il-1β* R: CTTGTGACCCTGAGCGACC | Sangon Biotech | Custom made |
| Mouse-*TNF-α* F: GATCGGTCCCCAAAGGGATG | Sangon Biotech | Custom made |
| Mouse-*TNF-α* R: TTTGCTACGACGTGGGCTAC | Sangon Biotech | Custom made |
| Mouse-*Cxcl10* F: TGGCTGGGATTCACCTCAAG | Sangon Biotech | Custom made |
| Mouse-*Cxcl10* R: CCGTTACTTGGGGACACCTT | Sangon Biotech | Custom made |
| Mouse-*Ifng* F: GGAGGAACTGGCAAAAGGATG | Sangon Biotech | Custom made |
| Mouse-*Ifng* R: GACCTGTGGGTTGTTGACCT | Sangon Biotech | Custom made |
| Mouse-*Gzmb* F: CAGGACAAAGGCAGGGGAGAT | Sangon Biotech | Custom made |
| Mouse-*Gzmb* R: AGGGATGACTTGCTGGGTCT | Sangon Biotech | Custom made |
| Mouse-*Ccl5* F: GCTGCTTTGCCTACCTCTCC | Sangon Biotech | Custom made |
| Mouse-*Ccl5* R: TCGAGTGACAAACACGACTGC | Sangon Biotech | Custom made |
| Mouse-*Gzma* F: GCTTATTCCTGAAGGAGGCTGT | Sangon Biotech | Custom made |
| Mouse-*Gzma* R: CAGCAGTCAACACCCAGTTC | Sangon Biotech | Custom made |
| Mouse-*Prf1* F: TTGGTGGGACTTCAGCTTTCC | Sangon Biotech | Custom made |
| Mouse-*Prf1* R: CCATACACCTGGCACGAACT | Sangon Biotech | Custom made |
| Universal 16S rRNA F: GCCAGCAGCCGCGGTAA | Sangon Biotech | Custom made |
| Universal 16S rRNA R: AGGGTATCTAATCCT | Sangon Biotech | Custom made |
| B. *Intestinalis* probe: CATTTGCCTTACGGCTAACCTG | Focobio | Custom made |
| EUB 338 probe: GCTGCCTCCCGTAGGAGT | Focobio | Custom made |
| NON 338 probe: ACATCCTACGGGAGGC | Focobio | Custom made |
| Human shATP5F1A： CCGGCCTCTGTTGATCTTGAAGAAACTCGAGTTTCTTCAAGATCAACAGAGGTTTTTG | Tsingke Biotech | Custom made |
| Human-*Atp5f1a* F: GTATTGCCCGCGTACATGG | Sangon Biotech | Custom made |
| Human-*Atp5f1a* R: AGGACATACCCTTTAAGCCTGA | Sangon Biotech | Custom made |
| **Software and Algorithms** |  |  |
| FlowJo10.4 | TreeStar | https://www.flowjo.com RRID: SCR_008520 |
| Prism 6 | GraphPad software | https://www.graphpad.com/ RRID: SCR_002798 |
| ImageJ | ImageJ | https://imagej.net/ RRID:SCR_003070 |
| UniProt database | N/A |  |
| **Deposited data** |  |  |
| 16S rRNA gene sequencing | SRA | NCBI Sequence Read Archive (SRA): PRJNA1313405 |
| Single cell sequencing | SRA | NCBI Sequence Read Archive (SRA): PRJNA1295121 |
| Untargeted metabolomics | Metabolomics Workbench | PR002551 |
